# The consequences of switching strategies in a two-player iterated survival game

**DOI:** 10.1007/s00285-021-01569-3

**Published:** 2021-02-06

**Authors:** Olivier Salagnac, John Wakeley

**Affiliations:** 1 École Normale Supérieure, Paris, Cedex 05, France; 2 Department of Organismic and Evolutionary Biology, Harvard University, Cambridge, MA 02138 USA

**Keywords:** Cooperation, Prisoner’s Dilemma, Iterated game, Survival game, 91A20, 91A05, 92D15, 92D50, 91A55

## Abstract

We consider two-player iterated survival games in which players are able to switch from a more cooperative behavior to a less cooperative one at some step of an *n*-step game. Payoffs are survival probabilities and lone individuals have to finish the game on their own. We explore the potential of these games to support cooperation, focusing on the case in which each single step is a Prisoner’s Dilemma. We find that incentives for or against cooperation depend on the number of defections at the end of the game, as opposed to the number of steps in the game. Broadly, cooperation is supported when the survival prospects of lone individuals are relatively bleak. Specifically, we find three critical values or cutoffs for the loner survival probability which, in concert with other survival parameters, determine the incentives for or against cooperation. One cutoff determines the existence of an optimal number of defections against a fully cooperative partner, one determines whether additional defections eventually become disfavored as the number of defections by the partner increases, and one determines whether additional cooperations eventually become favored as the number of defections by the partner increases. We obtain expressions for these switch-points and for optimal numbers of defections against partners with various strategies. These typically involve small numbers of defections even in very long games. We show that potentially long stretches of equilibria may exist, in which there is no incentive to defect more or cooperate more. We describe how individuals find equilibria in best-response walks among *n*-step strategies.

## Introduction

In a two-player iterated survival game, individuals may or may not survive each step and an individual whose partner has died must continue alone (Eshel and Weinshall 1988). It is a game against Nature (Lewontin 1961) such as when individuals have to fend off repeated attacks by a predator (Garay 2009; De Jaegher and Hoyer 2016) or face other sorts of adversity (Emlen 1982; Harms 2001; Smaldino et al. 2013; De Jaegher 2019). As Darwin (1859, p. 69) had noted: “When we reach the Arctic regions, or snow-capped summits, or absolute deserts, the struggle for life is almost exclusively with the elements.” Observing animals living together under harsh physical and biological conditions, Kropotkin (1902) suggested that mutual aid is inevitable in evolution. Iterated survival games are a simple way to model these scenarios, and they do show that self-sacrificing cooperative behaviors can be strongly favored when the prospects for lone individuals are not great (Eshel and Weinshall 1988; Eshel and Shaked 2001; Garay 2009; Wakeley and Nowak 2019).

We consider iterated survival games of fixed length *n*. We assume that there are two possible single-step strategies or behaviors: *C* and *D*. The probability an individual lives through a single step is given by Table 1, and the game is symmetric in that both players receive payoffs (live or die in each step) according to this matrix. We assume that $a>d$, so individuals in *CC* pairs fare better than individuals in *DD* pairs. Total payoffs, which are overall survival probabilities, accrue multiplicatively across the *n* steps. These depend on the overall, *n*-step strategies of individuals, which are fixed strings of *C*s and *D*s. We limit our attention to strategies which switch from the more cooperative behavior (*C*) to the less cooperative behavior (*D*) once, at some step of the game. Our goal is to understand the consequences of this, both for individual survival within a game and for overall strategy choice by individuals.

**Table 1:** The single-step payoff (*a*, *b*, *c*, *d* or $a_0$) in a symmetric two-player survival game is the probability of survival of an individual when the individual and partner have specified single-step strategies, either *C* or *D*, or when the individual is playing alone because the partner has died (Ø)

		Partner		
		*C*	*D*	Ø
Individual	*C*	*a*	*b*	$a_0$
	*D*	*c*	*d*	$a_0$

The loner survival probability, $a_0$, does not depend on the individual’s strategy. All five payoff values are strictly between 0 and 1

From the standpoint of behavioral biology or mathematical ecology, this is a phenomenological rather than a mechanistic model (Geritz and Kisdi 2012). It is described plainly in terms of the relative survival of types in different combinations, and skirts any details about ‘who helps whom achieve what’ (Rodrigues and Kokko 2016). Survival is an obviously crucial kind of utility for individuals, which also combines in various ways with fertility to produce evolutionary fitness (Argasinski and Broom 2013). Here, we do not consider differences in fertility. The only payoff is survival: one if the individual survives to the end of the game, zero otherwise. We allow any values between 0 and 1 for all five single-step payoffs ($a,b,c,d,a_0$) but we assume that they are fixed for the entire game and that survival outcomes are statistically independent, both in different steps and for different players in a single step. The consequent multiplicative accrual of payoffs turns relatively mild single-step games into mortally challenging iterated games as *n* increases. This naturally produces strong interdependence between individuals, which is known to favor cooperation and is purposely assumed in other models (Roberts 2005).

When both players are present, then depending on the magnitudes of *a* versus *c* and *b* versus *d*, each step will fall into one of the four well-known classes of symmetric two-player games. Ignoring the possibility that some payoffs might be equal: $a<c$ and $b<d$ defines the class of games represented by the Prisoner’s Dilemma (Tucker 1950; Rapoport and Chammah 1965); $a>c$ and $b<d$ defines the class represented by the Stag Hunt (Skyrms 2004); $a<c$ and $b>d$ defines the class represented by the Hawk-Dove game (Maynard Smith and Price 1973; Maynard Smith 1978); and $a>c$ and $b>d$ defines the class which was recently dubbed the Harmony Game (De Jaegher and Hoyer 2016). In the case of the Prisoner’s Dilemma, *a* corresponds to the “reward” payoff, *b* to the “sucker’s” payoff, *c* to the “temptation” payoff, and *d* to the “punishment” payoff (Rapoport and Chammah 1965).

Wakeley and Nowak (2019) considered individuals with constant strategies, all-*C* or all-*D*, and studied how the relative frequency of all-*C* changes over time in a well-mixed population due to differential death in the two-player iterated survival game. Depending especially on the number of iterations *n* and the loner survival probability $a_0$, the *n*-step game may be of a different type than the single-step game, with obvious implications for the evolution of cooperation. For example, if *n* is large and $a_0$ is small, the *n*-step game may be a Harmony Game even if the single-step game is a Prisoner’s Dilemma. Then cooperation is favored despite the fact that it seems better to defect in any given step. On the other hand, if $a_0$ is large, the *n*-step game may favor all-*D* even if the single-step game is a Harmony Game.

Here we study the problem of strategy choice for a broader range of *n*-step strategies, specifically ones which switch from *C* to *D* at some step of the game. Strategy $S_i$ plays *D* for the final *i* steps of the game (and *C* for the first $n-i$ steps). Thus, $S_0$ is all-*C* and $S_n$ is all-*D*. The series of single-step strategies between an individual with strategy $S_j$ and a partner with strategy $S_i$ may be depicted as

1 $$\begin{aligned} \begin{aligned} S_j&= \underbrace{CCC\dots C}_{n-j ~ \mathrm {steps}} \underbrace{DDD\dots D}_{j-i~ \mathrm {steps}}\underbrace{DDD\dots D}_{i~ \mathrm {steps}}\\ S_i&=\overbrace{CCC\dots C}_{} \overbrace{CCC\dots C}_{ }\overbrace{DDD\dots D}_{} \end{aligned} \end{aligned}$$

in the case $j \ge i$. Considering all $i,j\in \llbracket 0,n \rrbracket$, we ask how the individual’s probability of survival depends on *j* given *i*, as well as on the other six parameters $(a,b,c,d,a_0,n)$. We wish to understand how cooperation may be supported in these games. We describe the structure of incentives for increasing or decreasing the number of end-game defections, and we identify optima for which there is no incentive for the individual (or the partner) to change strategy.

We focus on the case where each single-step game is a Prisoner’s Dilemma and ask how the incentive to defect may be undermined upon iteration when loners are at a relative disadvantage. However, we describe these games over the full range of $a_0$ and present some results for all four classes of single-step games. Restricting attention to strategies of the form $S_i$ allows us to work with closed-form expressions for overall payoffs in a simple space of strategies. We assume that individuals possess one of the $n+1$ possible pure strategies and make choices among these based on overall payoffs. With respect to single-step Prisoner’s Dilemmas, we know that $S_1$ is favored over $S_0$ but we do not know how far back into the game such advantages extend. Although we do not consider mixed strategies or frequencies of strategies in populations, our results have immediate consequences for Nash equilibria and evolutionary stability (Hofbauer and Sigmund 1998; Cressman 2005; Sandholm 2010).

## Markov model of individual survival and preliminary calculations

The survival game is symmetric, so we can focus on one player, nominally the individual of Table 1. The individual is in one of three possible situations: alive with a partner, alive without a partner or dead. We use a Markov chain to model transitions among these three states. The probabilities of surviving to the next round are given by Table 1, symmetrically for both players, and players live or die independently of one another in each step of the game. The chain is non-homogenous because transition probabilities depend on the single-step strategies of the individual and partner in each round of the game, and these may change, for example as in (1).

There are four ways the individual can be alive with a partner, or four possible pairs of single-step strategies, with the individual listed first and the partner listed second: *CC*, *DC*, *CD*, and *DD*. We use these to index four corresponding single-step transition matrices. We use Ø   to denote that one of the players has died and $*$ as a placeholder for the partner when the individual has died. The game always starts with two players, but then changes state randomly according to these matrices.2345Note that the column labels in (2) through (5) denote the situation at the end of the current step of the game, before any switch from *C* to *D* occurs. The single-step strategies of the individual and partner in the next round of the game are as specified by their overall, *n*-step strategies $S_j$ and $S_i$.

**Fig. 1:**
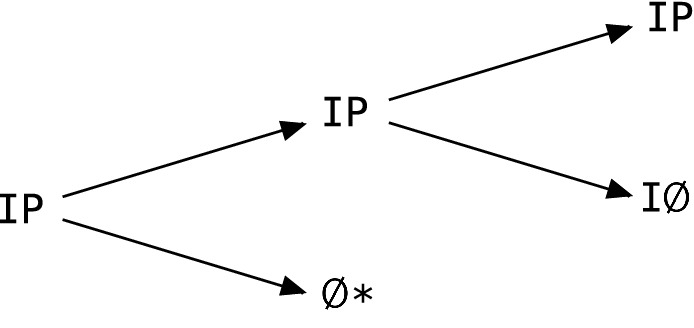
Two-event decomposition of a single step in the iterated survival game when both players are present, illustrating Individual-Partner dependence. The diagram can be used to compute the first-row transition probabilities in the matrices in (2) through (5) by replacing I and P with strategies *C* or *D* then assigning probabilities to the arrows

The second and third rows of all four matrices are identical due to our assumption of a single loner survival probability regardless of strategy, and because the state Ø$*$ is absorbing for the individual. The transitions described by the first rows of the matrices are more complex because they involve two events, one for the individual and one for the partner. Although payoffs are awarded simultaneously to both players in determining the transition probabilities in the first rows, this two-fold structure of the single-step game between two players lends itself to depiction as an extensive form game (von Neumann 1928; Kuhn 1953; Cressman 2005). This is illustrated in Fig. 1 and underscores the strong dependence between players in an iterated survival game. Figure 1 is also a probability tree diagram because the transition probabilities in the first rows in (2) through (5) can be obtained by multiplying probabilities associated with the arrows given specified single-step strategies *C* or *D*.

An individual with a partner may die, in which case the game is over for the individual regardless of what happens to the partner. This event is represented by the first down-arrow in Fig. 1. Having a large survival probability when the partner is present is the only protection against this fate for the individual. Here, the usual comparisons of *a* versus *c* and *b* versus *d* describe the consequence of switching strategies against a partner with a given strategy. However, the future state of the individual also depends on what happens to the partner. If the partner dies (second down-arrow in Fig. 1), the individual ends up alone and will be subject to the loner survival probability in every remaining step of the game.

The only way to remain in state one of the Markov chain is for both players to survive (both up-arrows in Fig. 1). The probability of this combined event is given by the upper-left or (1,1) entries in each matrix, which depend on the strategies of both players. Thus, the consequences of switching strategies will also depend on the comparisons of $a^2$ versus *bc* and *bc* versus $d^2$. This can be understood in terms of the number of cooperators in each possible pair of single-step strategies. Switching from *D* to *C* against a *D* partner changes the number of cooperators in the pair from zero to one, and switching from *D* to *C* against a *C* partner changes it from one to two. The inclusion of the first cooperator in a pair has effect $bc-d^2$ whereas the inclusion of a second cooperator has effect $a^2-bc$. Then, for example, an individual who suffers a cost $b-d<0$ in a Prisoner’s Dilemma might also enjoy the benefit of not having to survive alone, if it is also true that $bc-d^2>0$.

Our goal is to understand the overall survival probability of an individual whose strategy is $S_j$ given a partner with strategy $S_i$ for all $i,j\in \llbracket 0,n \rrbracket$. Any such game can be partitioned into three phases: both players having strategy *C*, one *C* and one *D*, and both *D*. The ordered series of these will determine the overall transition matrix. For the example in (1), we have the product $A^{n-j}_{CC} A^{j-i}_{DC} A_{DD}^{i}$.

We employ the following decomposition—exemplified by the case *CC*, when both players having strategy *C*—in order to compute the powers of the four matrices.

6 $$\begin{aligned} A_{CC}&= \begin{pmatrix}a^2 &{} a(1-a) &{} 1-a \\ 0 &{} a_0 &{} 1-a_0 \\ 0 &{} 0 &{} 1 \end{pmatrix} \nonumber \\&=\begin{pmatrix}1&{}a(1-a)&{}1\\ 0&{}a_0 -a^2 &{}1\\ 0&{}0&{}1\end{pmatrix} \begin{pmatrix}a^2 &{}0&{}0\\ 0&{}a_0&{}0\\ 0&{}0&{}1\end{pmatrix} \begin{pmatrix}1&{}\frac{a(a-1)}{a_0 - a^2} &{} \frac{a-a_0}{a_0 -a^2} \\ 0&{} \frac{1}{a_0 -a^2} &{} \frac{-1}{a_0 -a^2} \\ 0&{}0&{}1\end{pmatrix} . \end{aligned}$$

The diagonal elements in the middle matrix in (6) and in $A_{CC}$ itself are the eigenvalues of $A_{CC}$. The two outer matrices in (6) are the inverses of each other. Then for any number of steps, $k = 0,1,2,\ldots$, we have

7 $$\begin{aligned} A_{CC}^k&= \begin{pmatrix}1&{}a(1-a)&{}1\\ 0&{}a_0 -a^2 &{}1\\ 0&{}0&{}1\end{pmatrix} \begin{pmatrix} a^{2k} &{}0&{}0 \\ 0&{}a_0^k&{}0 \\ 0&{}0&{}1 \end{pmatrix} \begin{pmatrix}1&{}\frac{a(a-1)}{a_0 - a^2} &{} \frac{a-a_0}{a_0 -a^2} \\ 0&{} \frac{1}{a_0 -a^2} &{} \frac{-1}{a_0 -a^2} \\ 0&{}0&{}1\end{pmatrix} \end{aligned}$$

8 $$\begin{aligned}&=\begin{pmatrix}a^{2k}&{} a(1-a)\frac{a_0^k-a^{2k}}{a_0 -a^2} &{} 1-a^{2k}-a(1-a)\frac{a_0^k - a^{2k}}{a_0 - a^2} \\ 0 &{} a_0^k &{} 1-a_0^k \\ 0&{}0&{}1\end{pmatrix} . \end{aligned}$$

Applying the same technique to $A_{DC}$, $A_{CD}$ and $A_{DD}$ we obtain

9 $$\begin{aligned}&A_{DC}^{k} =\begin{pmatrix}(bc)^{k}&{} c(1-b)\frac{a_0 ^{k}-(bc)^{k}}{a_0 -bc} &{} 1-(bc)^{k}-c(1-b)\frac{a_0 ^{k}-bc^{k}}{a_0 -bc} \\ 0 &{} a_0 ^{k} &{} 1-a_0 ^{k} \\ 0&{}0&{}1\end{pmatrix} \end{aligned}$$

10 $$\begin{aligned}&A_{CD}^{k} =\begin{pmatrix}(bc)^{k}&{} b(1-c)\frac{a_0 ^{k}-(bc)^{k}}{a_0 -bc} &{} 1-(bc)^{k}-b(1-c)\frac{a_0 ^{k}-bc^{k}}{a_0 -bc} \\ 0 &{} a_0 ^{k} &{} 1-a_0 ^{k} \\ 0&{}0&{}1\end{pmatrix} \end{aligned}$$

11 $$\begin{aligned}&A_{DD}^k =\begin{pmatrix}d^{2k}&{} d(1-d)\frac{a_0^k - d^{2k}}{a_0-d^2} &{} 1-d^{2k}-d(1-d)\frac{a_0^k-d^{2k}}{a_0-d^2} \\ 0 &{} a_0^k &{} 1-a_0^k \\ 0&{}0&{}1\end{pmatrix} . \end{aligned}$$

Note that some quotients in (8) through (11) and in many payoff functions which follow are indeterminate for specific choices of single-step payoffs, for example if $a_0=d^2$ in (11). However, these terms represent sums of geometric series which are well-defined for all payoff values.

With these preliminary calculations, we can determine the *n*-step payoff of $S_j$ versus $S_i$, which will be the focus of our analysis. We call this payoff $A(S_j;S_i)$ and note that it is equal to the probability the individual with strategy $S_j$ is still alive after the *n* steps of the game. For the case $j \ge i$, we have

12 $$\begin{aligned} A(S_j;S_i) =&~ (A_{CC}^{n-j} A_{DC}^{j-i} A_{DD}^i)_{(1,1)}+(A_{CC}^{n-j} A_{DC}^{j-i} A_{DD}^i)_{(1,2)} \nonumber \\ =&~ a^{2(n-j)}(bc)^{j-i}d^{2i} + a(1-a)a_0^j \frac{a_0^{n-j}-a^{2(n-j)}}{a_0-a^2}\nonumber \\&~ + c(1-b)a^{2(n-j)}a_0^{i}\frac{a_0^{j-i} - (bc)^{j-i}}{a_0-bc}\nonumber \\&+d(1-d)a^{2(n-j)}(bc)^{j-i} \frac{a_0^i-d^{2i}}{a_0 -d^2} . \end{aligned}$$

For the case where $j \le i$, we get the symmetric result in *b* and *c*, as well as in *i* and *j*,

13 $$\begin{aligned} A(S_j;S_i) =&~ (A_{CC}^{n-i} A_{CD}^{i-j} A_{DD}^j)_{(1,1)}+(A_{CC}^{n-i} A_{CD}^{i-j} A_{DD}^j)_{(1,2)} \nonumber \\ =&~ a^{2(n-i)}(bc)^{i-j}d^{2j} + a(1-a)a_0^i \frac{a_0^{n-i}-a^{2(n-i)}}{a_0-a^2} \nonumber \\&~ + b(1-c)a^{2(n-i)}a_0^{j}\frac{a_0^{i-j}-(bc)^{i-j}}{a_0-bc}\nonumber \\&+d(1-d)a^{2(n-i)}(bc)^{i-j} \frac{a_0^j-d^{2j}}{a_0 -d^2} . \end{aligned}$$

The four terms in (12) and (13) correspond to particular sub-events: the first is when the partner also stays alive during the whole game, while the remaining three are when the partner dies either when both players have strategy *C*, when one has *C* and one has *D*, or when both have *D*.

## Playing with a fully cooperative partner

Here we consider an individual with strategy $S_j$ and a partner with strategy $S_0$. We ask what strategy the individual should adopt to maximize survival given the specific game parameters $(a,b,c,d,a_0,n)$. We introduce methods which we extend to $S_j$ versus general $S_i$ in Sects. 4 and 5. In Sect. 3.1, we illustrate differences among the four well-known classes of (single-step) games, highlighting the importance of the loner survival probability $a_0$ in determining broad patterns of strategy choice in iterated survival games. In Sect. 3.2, we focus on the case in which the single-step game is a Prisoner’s Dilemma, and ask how far the notion of backward induction may be applied to iterated survival games.

The *n*-step payoff of $S_j$ against $S_0$ is obtained by putting $i=0$ in (12):

14 $$\begin{aligned} A(S_j;S_0)&= \frac{a-a^2}{a_0-a^2}a_0^n+a^{2n}\left[ \frac{a_0 -c}{a_0 -bc} \left( \frac{bc}{a^2}\right) ^j + \left( \frac{c-bc}{a_0 - bc}-\frac{a-a^2}{a_0 -a^2}\right) \left( \frac{a_0}{a^2}\right) ^j \right] . \end{aligned}$$

Thus, $A(S_j;S_0)$ depends on three individual survival probabilities (*a*, *b*, *c*), as well as on the pair survival probabilities $(a^2,bc)$ and the loner survival probability $(a_0)$ which are eigenvalues of the single-step matrices in (2) and (3). It does not depend on *d* because there are no steps in which both players use strategy *D*. The dependence on *n* is simple: $A(S_j;S_0)$ tends to zero as *n* tends to infinity. Surviving longer is always less likely. Conveniently for our purposes, $A(S_j;S_0)$ depends on *j* only through the terms in the brackets, which do not include *n*. We focus on these terms and treat *n* implicitly, noting of course that $j \le n$. Because the terms in brackets may increase as *j* increases, it should be noted that $A(S_j;S_0)$ is a probability—it can never exceed 1—and that if $j=n$ and *n* tends to infinity, $A(S_j;S_0)$ tends to zero.

We wish to know the value of $j \in \llbracket 0,n \rrbracket$ which maximizes the survival probability of the individual for a given parameters $(a,b,c,a_0)$. Although *j* is discrete, in order to find the optimum we treat (14) as a continuous function of $j\in [0,n]$. Three cases can occur, because there is at most one change in sign of the slope. The maximum can be reached when $j=0$, which would happen for example when $a^2>a_0>c$. Then the fully cooperative behavior has the greatest chance of survival, no matter how many rounds are being played. Alternatively, the supremum of the function may be in the limit $j\rightarrow \infty$. Then, for large enough *n*, the best *j* would be *n*. In this case $S_n$, or all-*D*, would have the greatest chance of survival against $S_0$. A third case is that the function has a maximum at some intermediate value, specifically at

15 $$\begin{aligned} j_{opt} = \frac{\ln {\left[ \left( \frac{a(1-a)(a_0-bc)}{(a_0-c)(a_0-a^2)}-\frac{c(1-b)}{a_0-c} \right) \frac{ \ln { \left( \frac{a_0}{a^2}\right) }}{\ln {\left( \frac{bc}{a^2} \right) }} \right] }}{\ln {\left( \frac{bc}{a_0}\right) }} \end{aligned}$$

which exists when the argument of the logarithm in the numerator is positive. In this case, there could be an intermediate step in the game which gives the greatest benefit of switching from *C* to *D*. The integer-valued optimum *j* would be one of the integers

16 $$\begin{aligned} J_{opt} = \left\{ \begin{array}{c}\left\lfloor j_{opt} \right\rfloor \\ \mathrm {or} \\ \left\lceil j_{opt} \right\rceil \end{array} \right. \end{aligned}$$

on either side of the real-valued $j_{opt}$. If $n \le j_{opt}$, then all-*D* ($S_n$) is the best strategy against all-*C* ($S_0$). If $n > j_{opt}$, then the optimal number of defections is $J_{opt}$ which does not depend on *n*. In the Prisoner’s Dilemma and the Hawk-Dove game, it will always be advantageous to defect in the final round of the game, because $c>a$. But when $j_{opt}$ exists, additional end-game defections will be favored only up to $J_{opt}$ even against a partner who commits to full cooperation in an arbitrarily long game.

### Comparison of the four types of games

**Fig. 2:**
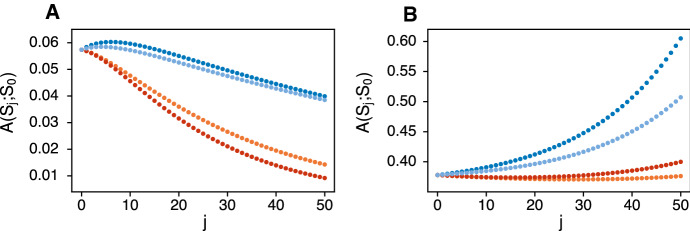
The probability of survival of an individual who switches strategy from *C* to *D* for the last *j* steps of an iterated survival game of length $n=50$ against an all-*C* partner. In **a** the loner survival probability is small, $a_0=0.8$, and in **b** the loner survival probability is large, $a_0=0.99$. Colors denote examples of the four possible kinds of games: blue is a Prisoner’s Dilemma ($a=0.97$, $b=0.94$, $c=0.99$, $d=0.95$), light blue is a Hawk-Dove game ($a=0.97$, $b=0.95$, $c=0.98$, $d=0.94$), orange is a Harmony Game ($a=0.97$, $b=0.95$, $c=0.96$, $d=0.94$), red is a Stag Hunt ($a=0.97$, $b=0.94$, $c=0.96$, $d=0.95$)

Figure 2 shows $A(S_j;S_0)$ as a function of *j* in a game of length $n=50$ for examples of the four classes of games, when the loner survival probability is either small (Fig. 2a) or large (Fig. 2b). The other payoffs (*a*, *b*, *c*, *d*) are the same in both panels. For the example Prisoner’s Dilemma, these payoffs ($a=0.97$, $b=0.94$, $c=0.99$, $d=0.95$) are a linear transformation of the classic payoffs ($R = 3$, $S = 0$, $T = 5$, $P = 1$) of Axelrod (1984). For all four games in Fig. 2A the relationship of the eigenvalues is $a^2> bc > a_0$. In Fig. 2b it is $a_0> a^2 > bc$. Again, we are interested in whether the highest survival occurs at one or the other extreme, $j=0$ or $j=n$, or at some intermediate $J_{opt}$. An optimal intermediate strategy exists in these examples only for the Prisoner’s Dilemma and the Hawk-Dove game with small $a_0$ (Fig. 2a). When $a_0$ is the smallest eigenvalue, there is a high cost to a player being alone for a long stretch. The optimal strategy balances the increased chance of paying this cost against the increase in survival from switching from *C* to *D* in the Prisoner’s Dilemma and the Hawk-Dove game. If, as in the Stag Hunt and Harmony Game in Fig. 2a, switching from *C* to *D* does not directly increase survival, then $S_0$ (all-*C*) is best.

On the other hand, when $a_0$ is large, a lone individual may have an advantage. This is true in all four examples in Fig. 2b, where $a_0$ is the largest individual payoff. For large *j*, the term in brackets in (14) is strictly increasing in *j*. Provided the game is long enough, $S_n$ (all-*D*) will be the best strategy in all four cases. Here, additional defections by the individual put the partner at greater risk because $b<a$ and $b<c$. But in the Harmony Game and the Stag Hunt it is also true that $c<a$, meaning that the individual pays a cost to put the partner at risk. This causes minima of survival at intermediate *j* in these two games. The individual only sees the benefits of the high loner payoff at larger *j* in longer games. The Prisoner’s Dilemma and the Hawk-Dove game do not show this dip in survival for small *j* because they both have $c>a$. Note that changing the level of risk to the partner in the Harmony Game and the Stag Hunt can drastically alter these results. For example, putting $b=0.98$ in this Harmony Game completely removes the risk to the partner, while preserving the order of eigenvalues and the fact that $a_0$ is the largest individual payoff. Now any increase in *j* will be disadvantageous because the term in brackets in (14) is negative.

Figure 2 reveals some key features and some complexities of strategy choice in iterated survival games. The four-fold classification of games based on the comparison of *a* to *c* and *b* to *d*, together with the rough criteria of large versus small $a_0$ is not enough to determine the potential advantages of switching strategies from *C* to *D* at some point in the game. The order of the eigenvalues $(a^2,bc,d^2,a_0)$ is crucial. The example games in Fig. 2 all have $a^2> bc > d^2$, but it could be otherwise. For some games, we might have $a^2> d^2 > bc$ and for others $bc> a^2 > d^2$. The assumption that *C* is the more cooperative and *D* the less cooperative strategy, hence $a>d$, guarantees that $a^2 > d^2$. But in all cases, $a_0$ could be anywhere in the order of eigenvalues. In what follows, we focus on the classic challenge to cooperation, the Prisoner’s Dilemma of Tucker (1950) and Rapoport and Chammah (1965). This is a restricted version of what we have been calling the Prisoner’s Dilemma class of games, specifically satisfying conditions (17) and (18) below. Our aim is to determine in detail when a late defection might be optimal or when an early one would be better, depending especially on the magnitude of the loner survival probability, $a_0$.

### Defection against a fully cooperative partner in the Prisoner’s Dilemma

When the single-step game is a Prisoner’s Dilemma, playing *D* in the final step of an *n*-step game will always increase the survival probability of an individual. If payoffs accrued additively as in the classical repeated Prisoner’s Dilemma (Rapoport and Chammah 1965; Axelrod 1984) then by backward induction the same logic would apply to every preceding step of the game. Seeing an uninterrupted sequence of increased chances of survival, an all-*C* individual facing an all-*C* partner would switch to all-*D*. But payoffs do not accrue additively in an iterated survival game. We have already established that an optimal number of defections, $J_{opt}$ in (16), may exist. Here we study in detail how this depends on the loner survival probability.

We make use of the classical assumptions of the Prisoner’s Dilemma, described for example by Rapoport and Chammah (1965, p. 34):

17 $$\begin{aligned}&c> a> d > b , \end{aligned}$$

18 $$\begin{aligned}&a> (b+c)/2 \implies a^2 > bc . \end{aligned}$$

The broader class of games which includes this Prisoner’s Dilemma is defined just by $c>a$ and $d>b$. Again, $a > d$ in (17) guarantees that $a^2 > d^2$, so the survival probability of the pair is higher when both players cooperate than when both defect. The additional restriction to $a^2 > bc$ in (18) means that pairs survive better when both players cooperate than when just one player cooperates. This is not a major restriction, as 90% of the parameter space of survival games $(0<a,b,c,d<1)$ for which (17) is satisfied also has $a^2 > bc$ (Wakeley and Nowak 2019). Note that (17) and (18) do not determine the relationship between *bc* and $d^2$.

We base our detailed analysis on the payoff difference

19 $$\begin{aligned}&A(S_j;S_0)-A(S_0;S_0) \nonumber \\&\qquad = a^{2n} \left[ \frac{a_{0} - c}{ a_{0}-bc} \left( \frac{bc}{a^2} \right) ^{j} + \left( \frac{c(1-b)}{a_0 -bc} -\frac{a(1-a)}{a_0 -a^2} \right) \left( \frac{a_0}{a^2} \right) ^{j} \right. \left. - \frac{a_0-a}{a_0 - a^2} \right] . \end{aligned}$$

When this difference is positive, there is incentive for an individual currently playing all-*C* against an all-*C* partner to switch strategies and defect for the final *j* rounds of the game. When it is negative, the individual is better off sticking with all-*C*, or $S_0$. The *j* for which this difference is the largest will be the optimal number of end-game defections given a fully cooperative partner.

As in (14), there is a separation of *n* and *j*. The same two exponential terms are present within the brackets, which will increase, decrease or remain constant as *j* increases, depending on the ratios of eigenvalues, $bc/a^2$ and $a_0/a^2$. Again, the slope changes sign at most once. It is straightforward to compute $A(S_0;S_0)-A(S_0;S_0)=0$ and $A(S_1;S_0)-A(S_0;S_0)=a^{2(n-1)}(c-a)>0$. Then for the Prisoner’s Dilemma (i.e. with $c>a$), the payoff difference increases with *j* when *j* is small. The question is whether it continues to increase or reaches a peak and starts to decrease as *j* grows. Owing to (18), $bc/a^2$ is strictly less than one. But the parameter $a_0$ is free to vary between 0 and 1, so $a_0/a^2$ may be less than, greater than, or equal to one.

If $a_0 < a^2$, then both exponential terms in (19) will be decreasing in *j* and will eventually go to zero. At some point as *j* increases, assuming *n* is large enough, the difference $A(S_j;S_0)-A(S_0;S_0)$ will turn negative and converge to the constant $- a^{2n} (a_0-a)/(a_0 - a^2)$. Too many defections will ultimately hurt the player because the loner survival probability is small. Again, defecting just once at the end of the game is always favored because $c>a$. Therefore an optimal strategy will exist, with $J_{opt}$ given by (15) and (16). But if *n* is not large enough, then *j* will always be less than this optimum and the best strategy against $S_0$ will be $S_n$.

Instead if $a_0 > a^2$, then the difference $A(S_j;S_0)-A(S_0;S_0)$ will eventually be dominated by the middle term in (19). Depending on the sign of this term, $A(S_j;S_0)-A(S_0;S_0)$ will be increasing or decreasing when *j* is large. As there is at most one change in sign of the slope and the initial slope is positive, either the best strategy is complete defection or there exists an optimal intermediate strategy. The first occurs if and only if the middle term in (19) is positive. This induces a cutoff for $a_0$ as it varies between $a^2$ and 1. There is a shift in the behavior of $A(S_j;S_0)-A(S_0;S_0)$ as *j* increases, from having an intermediate optimum to always increasing, at

20 $$\begin{aligned} a^{*}_0 = \frac{c-a}{c-a + a^2-bc} a^2 + \frac{a^2-bc}{c-a + a^2-bc} a . \end{aligned}$$

The cutoff $a^{*}_0$ is the largest value of $a_0$ such that full defection might not be favored (i.e. there is a finite optimum *j*) against a fully cooperative partner. Again, if $n \le j_{opt}$, then full defection would still be the best strategy, even if $a_0<a^{*}_0$. But if $a_0>a^{*}_0$, then full defection will always be favored, for any *n*.

**Fig. 3:**
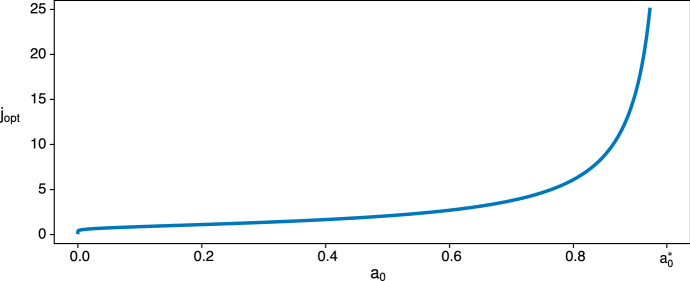
The optimal, real-valued point of defection $j_{opt}$ increases without bound as $a_0$ approaches $a^{*}_0$, for the same single-step Prisoner’s Dilemma game in Fig. 2, i.e. with $a=0.97$, $b=0.94$, $c=0.99$, $d=0.95$

The two survival differences which determine the coefficients of $a^2$ and *a* in (20) can be understood with reference to Fig. 1 and (2) and (3). The first, $c-a>0$, is the classic change in payoff for defecting against a cooperative partner, which here is the difference in the single-step survival probability of the individual regardless of what happens to the partner. The second, $a^2-bc>0$, expresses as a positive term the difference in the probability that both the individual and the partner survive. It is a single-step cost in pair survival but may be either a cost or a benefit to the individual depending on the values of $a_0$ and *n*. The coefficients in (20) sum to one, so the cutoff $a^{*}_0$ is an average falling between $a^2$ and *a*.

The cutoff $a^{*}_0$ is closer to $a^2$, and therefore smaller, when the benefit in individual survival, $c-a$, is large relative to the cost in pair survival, $a^2-bc$. When this is true, even a fairly small value of the loner survival probability $a_0$ cannot prevent $S_n$ from being the best strategy against $S_0$. On the other hand, $a^{*}_0$ is closer to *a*, and therefore larger, when the cost in pair survival is relatively big. When this is true, there may be an intermediate optimum strategy even when the loner survival probability is fairly large. Taking derivatives of $a^{*}_0$ provides some intuition about the effects of changing specific parameters, when other parameters are held constant. As long as the assumptions in (17) continue to be met, $a^{*}_0$ increases as *a* increases, but decreases when either *b* or *c* increases. In addition, if *b* increases and *c* decreases, together at the same rate so that *bc* approaches $a^2$, then $a^{*}_0$ will decrease toward $a^2$.

So far, we have considered two possibilities: $a_0 < a^2$ and $a_0 > a^2$. In the first case, $a^2$ is the largest eigenvalue, so a pair of cooperators survives a single step of the game better than any other pair and better than a lone individual. In this case, both terms that depend on *j* in (19) decrease to zero and the payoff difference $A(S_j;S_0)-A(S_0;S_0)$ converges to a finite negative constant, so there exists an optimum number of end-game defections, $J_{opt}$ in (16). In the second case ($a_0 > a^2$), a lone individual survives a single step better than any pair of individuals. But even when this is true, continuing to increase the number of end-game defections is advantageous only when $a_0$ exceeds $a^{*}_0$, which is larger than $a^2$. If $a^2< a_0 < a^{*}_0$, there is a $J_{opt}$ which may be relevant depending on the total number of steps in the game, *n*. Note that when $a_0 = a^{*}_0$ there is still a growing interest in defecting, but the dependence on *j* is different because the middle term in (19) is equal to zero and $A(S_j;S_0)-A(S_0;S_0)$ converges to a positive constant, $a^{2n} (c-a)/(a^2-bc)$, as *j* increases.

In the special case that $a^2=a_0$, we cannot use the results for geometric series which gave (8) through (11). Here we have

21 $$\begin{aligned} A(S_j;S_0)&=a^{2n}\left[ \frac{a^2-c}{a^2-bc}\left( \frac{bc}{a^2}\right) ^{j}+(n-j)\frac{1-a}{a}+\frac{c-bc}{a^2 -bc} \right] , \end{aligned}$$

22 $$\begin{aligned} \frac{\mathrm {d}}{\mathrm {d} j} A(S_j;S_0)&=a^{2n}\left[ \frac{a^2-c}{a^2-bc}\ln {\left( \frac{bc}{a^2}\right) }\left( \frac{bc}{a^2}\right) ^{j}-\frac{1-a}{a}\right] . \end{aligned}$$

As $bc<a^2<c$, the derivative will ultimately become negative, so there will be some optimal point of defection. Thus, $a_0=a^2$ is not pathological and belongs to the case $a^2< a_0 < a^{*}_0$. For technical reasons we distinguished three cases ($a_0 < a^2$, $a^2 \le a_0 < a^{*}_0$, $a^{*}_0 \le a_0$) but the important point is whether an optimum exisits. For this we have just two cases: $J_{opt}$ exists when $a_0 < a^{*}_0$ and does not exist when $a^{*} \le a_0$.

We turn now to the question of how $j_{opt}$ and $J_{opt}$ depend on $a_0$ when $a_0 < a^{*}_0$. Because larger $a_0$ indicates a smaller cost of being alone, it is intuitive that both quantities should increase with $a_0$. Figure 3 displays this for $j_{opt}$ and suggests that both $j_{opt}$ and $J_{opt}$ are increasing functions of $a_0$. Examination of $j_{opt}$ in (15) when $a_0$ is close to either of the extremes, 0 or $a^{*}_0$, gives

23 $$\begin{aligned} j_{opt} \underset{a_0 \rightarrow {} 0 }{\sim } \frac{\ln {\left( \frac{1-a}{a} \frac{\ln {\left( \frac{1}{a_0}\right) }}{\ln {\left( \frac{bc}{a^2} \right) }}\right) }}{\ln {\left( \frac{1}{a_0} \right) } } \underset{a_0 \rightarrow {} 0}{\rightarrow {}} 0 \end{aligned}$$

and

24 $$\begin{aligned} j_{opt} \underset{a_0 \rightarrow {} a^{*}_{0} }{\sim } \frac{\ln { \left( \frac{ a^{*}_{0} -a_0}{(a^{*}_0 -c)(a^{*}_0 - a^2)} \frac{\ln { \left( \frac{a^{*}_0}{a^2} \right) }}{\ln {\left( \frac{bc}{a^2} \right) }} \right) } }{\ln {\left( \frac{bc}{a^{*}_0 } \right) }} \underset{a_0 \rightarrow {}{a^{*}_0} }{\rightarrow } + \infty . \end{aligned}$$

For $J_{opt}$, using (16) and $A(S_1;S_0)-A(S_{0};S_{0}) = a^{2n}(c-a)>0$, we have

25 $$\begin{aligned} J_{opt} \underset{a_0 \rightarrow 0}{\rightarrow } 1 \end{aligned}$$

and

26 $$\begin{aligned} J_{opt} \underset{a_0 \rightarrow a^{*}_0}{\rightarrow } + \infty . \end{aligned}$$

In Appendix 1, we prove that $J_{opt}$ increases with $a_0$ for $a_0 < a^{*}_0$. Beyond this point, i.e. for $a_0 \ge a^{*}_0$, we may also say that $J_{opt}$ is infinite because regardless of *n* it will always be beneficial to increase the number of defections.

## Behavioral equilibria

Here we lift the restriction that the partner is fully cooperative, and ask whether there is an incentive to defect more or to cooperate more when the partner has strategy $S_i$. We will assume that $A(S_j;S_i) \ne A(S_i;S_i)$ unless $j=i$, both for simplicity and because we imagine $A(S_j;S_i) = A(S_i;S_i)$ to be unlikely in our model. Since the number of possible strategies $\{S_j$ ; $j \in \llbracket 0,n \rrbracket \}$ is finite, there will always be an optimal one against $S_i$. We are interested in identifying stable strategies, such that the individual cannot increase their probability of survival against a partner who has the same strategy. Strategy $S_i$ is optimal in this sense when

27 $$\begin{aligned} \forall j \ne i,~A(S_i;S_i) > A(S_j;S_i) . \end{aligned}$$

Then $S_i$ is a strict Nash equilibrium and therefore an evolutionarily stable strategy, or ESS (Maynard Smith and Price 1973; Maynard Smith 1982; Hofbauer and Sigmund 1998; Cressman 2005). A full account of Nash equilibria and ESSs would require the analysis of $A(S_j;S_i) = A(S_i;S_i)$ which we do not pursue here.

Due to (12) and (13), the cases $j>i$ and $j<i$ must be analyzed separately. Also, note there may be many equilibrium strategies. In this section we focus on local equilibria, meaning that the only options open to the individual are to defect one more time or cooperate one more time. Strategy $S_i$ is locally stable if and only if

28 $$\begin{aligned}&A(S_i;S_i) > A(S_{i+1};S_i) , \end{aligned}$$

29 $$\begin{aligned}&A(S_i;S_i) > A(S_{i-1};S_i) \end{aligned}$$

for $i \in \llbracket 1,n-1 \rrbracket$, with just (28) and (29) respectively at the endpoints $i=0$ and $i=n$. In Sect. 5, we consider global equilibria, for $A(S_j;S_i)$ over all $i,j \in \llbracket 0,n \rrbracket$.

### General results

We base our analysis of local stability on the two key differences

30 $$\begin{aligned}&A(S_{i+1};S_i)-A(S_i;S_i) \nonumber \\&\qquad = a^{2(n-1)} \left[ (bc-a^2) \frac{a_0 -d}{a_0 -d^2} \left( \frac{d^2}{a^2}\right) ^i + \left( (a^2 -bc) \frac{a_0 -d}{a_0 -d^2}+c-a\right) \left( \frac{a_0}{a^2}\right) ^i\right] \end{aligned}$$

31 $$\begin{aligned}&A(S_{i-1};S_i)-A(S_i;S_i) \nonumber \\&\qquad = a^{2(n-1)} \left[ (bc-d^2) \frac{a_0 -d}{a_0 -d^2} \left( \frac{d^2}{a^2}\right) ^{i-1} + \left( (d^2 -bc) \frac{a_0 -d}{a_0 -d^2}+b-d\right) \left( \frac{a_0}{a^2}\right) ^{i-1}\right] . \end{aligned}$$

Similar to (19), these two formulas show a separation of *i* and *n*. Their signs may depend on *i* but will not depend on *n*. Both formulas are sums of two exponential functions in *i*, with coefficients that depend on the game parameters $(a,b,c,d,a_0)$. They can change sign at most once. Therefore, the conditions for local stability in (28) and (29) will each be met—corresponding, respectively, to (30) and (31) being negative—either for a stretch of *i* or for no values of *i*. The set of locally stable *i* is the intersection of these two (possibly empty) stretches. In the case of defecting one more time, the stretch may range from 0 to $+\infty$. In the case of cooperating one more time, it may range from 1 to $+\infty$. Then, the locally stable strategies are a stretch of integers whose boundaries range from 1 to $+\infty$ (which may be empty) plus possibly 0. For the smallest *i*, (30) and (31) reduce to

32 $$\begin{aligned}&A(S_1;S_0)-A(S_0;S_0)=a^{2(n-1)}(c-a) , \end{aligned}$$

33 $$\begin{aligned}&A(S_0;S_1)-A(S_1;S_1)=a^{2(n-1)}(b-d) . \end{aligned}$$

Strategy $S_0$, or all-*C*, is locally stable if and only if $c<a$ which means that the single-step game is either a Harmony Game or a Stag Hunt (cf. Table 1). As in Sect. 3, we treat *n* implicitly in what follows, keeping in mind that any stretch of equilibria will depend on *n* in that *n* fixes the upper boundary of the interval. Our primary concern is to understand how the stretch of locally stable states depends on the other game parameters, in particular the loner survival probability $a_0$.

### Focusing on the Prisoner’s Dilemma

As in Sect. 3.2 we focus on the Prisoner’s Dilemma. Again we assume (17) and (18), namely that $c> a> d > b$ and $a^2 > bc$. In the following subsections, we first study the incentives (or disincentives) to either defect more or cooperate more, then consider the overlap of these two sets of results in order to identify equilibria, and finally provide a summary and interpretation of outcomes.

#### Incentives to defect more or cooperate more against $S_i$

Under the assumption that the single-step game is a Prisoner’s Dilemma, we have

34 $$\begin{aligned}&A(S_1;S_0)-A(S_0;S_{0})=a^{2(n-1)}(c-a) > 0 ,\ \end{aligned}$$

35 $$\begin{aligned}&A(S_0;S_1)-A(S_1;S_1)=a^{2(n-1)}(b-d) < 0. \end{aligned}$$

Thus, $i=0$ is never locally stable state when the single-step game is a Prisoner’s Dilemma. The difference $A(S_{i+1};S_i)-A(S_i;S_i)$ in (30) starts off positive for small *i* and will change sign at most once. We define the real-valued cutoff $i_D$ to be the point at which defecting one more time becomes disadvantageous as *i* increases. If (30) never changes sign, then $i_D$ does not exist and additional defection is always favored. When $i>i_D$, the strategy $S_i$ is a candidate for locally stability. Similarly, since $A(S_{i-1};S_i)-A(S_i;S_i)$ in (31) starts off negative for small *i* and changes sign at most once, we define $i_C$ to be the point at which increased cooperation first becomes advantageous. Here too $i_C$ may not exist. When $i<i_C$, the second criterion for local stability of strategy $S_i$ is met. Both criteria (28) and (29) are satisfied when $i \in \llbracket \lceil i_D \rceil ,\lfloor i_C \rfloor \rrbracket$, but this interval will be empty if $\lceil i_D \rceil > \lfloor i_C \rfloor$.

We begin with the case of increasing defection. If $a_0<d^2$, then $A(S_{i+1};S_i)-A(S_i;S_i)$ in (30) will ultimately become negative because the first term inside the brackets will come to dominate as *i* grows and this term is negative owing to our assumption that $a^2>bc$. If $a_0>d^2$, then (30) will ultimately become negative if and only if $(a^2 -bc) \frac{a_0 -d}{a_0 -d^2} +c-a <0$. Analogous to the situation in Sect. 3.2 with the cutoffs $a^{*}_0$ and $j_{opt}$, here we require

36 $$\begin{aligned} a_0 < a^{\prime }_0 = \frac{c-a}{c-a + a^2 -bc} d^2 + \frac{a^2 -bc}{c-a + a^2 -bc} d \end{aligned}$$

and find an associated cutoff for *i*

37 $$\begin{aligned} i_D=\frac{\ln {\left( 1+\frac{c-a}{a^2 -bc}\frac{a_0 -d^2}{a_0-d}\right) }}{\ln {\left( \frac{d^2}{a_0}\right) }} \end{aligned}$$

which exists if $a_0<a^\prime _0$. There is an advantage to defecting one more time only when $i<i_D$. For larger *i* it is disadvantageous. However, if $a_0 \ge a^\prime _0$, then $i_D$ does not exist and defecting one more time is advantageous for all *i*.

In the special case $a_0=d^2$, we obtain

38 $$\begin{aligned} A(S_{i+1};S_i)-A(S_i;S_i)=a^{2n}\left[ c-a+(bc-a^2)\frac{1-d}{d} i\right] \left( \frac{d^2}{a^2}\right) ^i \end{aligned}$$

which starts off positive for $i=0$ then turns negative for some larger *i*. Thus $a_0=d^2$ is not a pathological case but belongs with $a_0<d^2$ and $d^2<a_0<a^\prime _0$.

Like $a^*_0$ in (20), the cutoff $a^\prime _0$ in (36) is an average. Previously *i* was the number of defections the individual was considering against an all-*C* partner. Here *i* is the fixed number of *DD* rounds the individual must face when considering whether to defect one more time against an $S_i$ partner. As a result, $a^\prime _0$ is an average falling between $d^2$ and *d* instead of between $a^2$ and *a*. However, the coefficients determining where it falls are the same as before because the individual is making the same switch, from *C* to *D* when the partner has strategy *C* in that step. Thus, $a^\prime _0$ is closer to $d^2$, i.e. smaller, when the resulting gain in individual survival ($c-a$) is large relative to the loss in pair survival ($a^2-bc$), and closer to *d*, i.e. larger, when the opposite is true.

**Fig. 4:**
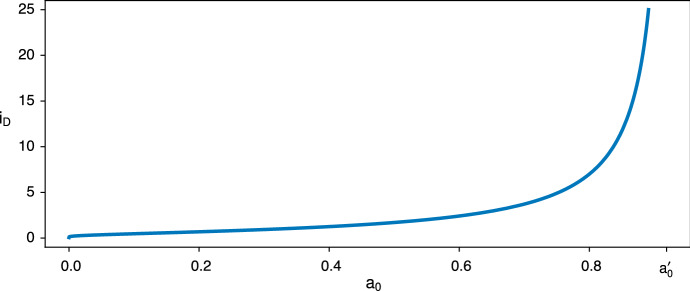
$i_D$ is the point above which defecting once more would become disadvantageous. It increases with $a_0$ toward $+\infty$ as $a_0$ approaches $a^{\prime }_0$. The parameters here are the same as in Fig. 3 ($a=0.97$, $b=0.94$, $c=0.99$, $d=0.95$)

Figure 4 illustrates that $i_D$ is an increasing function of $a_0$, growing from 0 to $+\infty$ as $a_0$ goes from 0 to $a^\prime _0$. As before, this fits with intuition about the balance between the benefit of defecting while the partner is still alive and the drawback of having to survive alone. The bigger $a_0$ is, the smaller this drawback becomes. The extremes of $i_D$ can be obtained from (37). We find

39 $$\begin{aligned}&i_D \underset{a_0 \rightarrow 0}{\rightarrow } 0 , \end{aligned}$$

40 $$\begin{aligned}&i_D \underset{a_0 \rightarrow a^{\prime }_0}{\rightarrow } +\infty . \end{aligned}$$

In Appendix 2, we prove that $i_D$ is indeed an increasing function of $a_0$.

Turning to the case of increasing cooperation, recall that $A(S_{i-1};S_i)-A(S_i;S_i)$ in (31) is negative for the smallest value, $i=1$. Additional cooperations will continue to be disfavored unless $A(S_{i-1};S_i)-A(S_i;S_i)$ changes sign and becomes positive at some $i_C$. If $i_C$ exists, then for any larger *i* it will be advantageous for the individual to cooperate one more time. Then for all $i>i_C$, strategy $S_i$ cannot be locally stable, whereas for $i<i_C$ it might be locally stable. Note that if the individual changes strategy from $S_i$ to $S_{i-1}$ against an $S_i$ partner, the pair-survival probability changes from $d^2$ to *bc*, and the individual survival probability changes from *d* to *b*. The net effect of the latter is negative ($b-d<0$). This direct disadvantage to additional cooperation may be offset by increased pair survival, but only if $bc>d^2$. Again, the assumptions in (17) and (18) do not determine the relationship of *bc* to $d^2$.

When $bc \le d^2$, the sign of $A(S_{i-1};S_i)-A(S_i;S_i)$ never changes because the net effect on pair survival, $bc-d^2$, is at most zero and will not be able to offset the direct, individual disadvantage of cooperating one more time. In this case $i_C$ does not exist, so all strategies are candidates for local stability, the upper limit being set only by *n*. When $bc > d^2$, the sign of the payoff difference may change, giving a finite $i_C$, but this will depend on the loner survival probability. If $a_0<d^2$, then $A(S_{i-1};S_i)-A(S_i;S_i)$ will eventually become positive. The case $a_0=d^2$ gives the same result, but is necessary again to compute the difference in probability without using the results for geometric series as we did previously for the condition on $A(S_{i-1};S_i)-A(S_i;S_i)$. If instead $a_0>d^2$, the payoff difference will ultimately become positive if and only if $(bc-d^2 ) \frac{a_0 -d}{a_0 -d^2} +d-b >0$. Overall, additional cooperation is favored when

41 $$\begin{aligned} a_0 < a^{\prime \prime }_0 = \frac{d-b}{d-b + bc - d^2} d^2 + \frac{bc-d^2}{d-b + bc - d^2 } d \end{aligned}$$

but only when *i* is greater than

42 $$\begin{aligned} i_C = 1 + \frac{\ln {\left( 1+\frac{d-b}{bc - d^2}\frac{a_0 -d^2}{a_0-d}\right) }}{\ln {\left( \frac{d^2}{a_0}\right) }} . \end{aligned}$$

Even when the loner survival probability is small, it will be disadvantageous to cooperate one more time if $i<i_C$. Using an approach like the one for $i_D$ in Appendix 2, it can be shown that $i_C$ is an increasing function of $a_0$ in the interval $(0,a^{\prime \prime }_0)$. At the extremes of loner survivability, we have

43 $$\begin{aligned}&i_C \underset{a_0 \rightarrow 0}{\rightarrow } 1 , \end{aligned}$$

44 $$\begin{aligned}&i_C \underset{a_0 \rightarrow a^{\prime \prime }_0}{\rightarrow } +\infty . \end{aligned}$$

Intuitively, the larger $a_0$ is, the lower the danger of a long stretch of mutual defection, so the individual is less inclined to risk a low probability of individual survival (*b*) in a given step for a greater chance of pair survival (*bc*). As $a_0$ approaches $a^{\prime \prime }_0$, surviving alone no longer becomes a drawback as *i* increases.

#### Stretches of locally stable strategies

The stretch of locally stable strategies is the interval of integers which satisfy the two conditions, (28) and (29). This interval is $\llbracket \lceil i_D \rceil ,\lfloor i_C \rfloor \rrbracket$ but is empty when $\lceil i_D \rceil > \lfloor i_C \rfloor$. There are three different cases to consider. The first case is $d^2 \ge bc$, such that $i_C$ does not exist regardless of $a_0$. With an upper limit of *n*, the integer interval begins as $\llbracket 1,n \rrbracket$ when $a_0$ is close to 0, then shrinks to an empty set as $a_0$ increases, because the lower boundary, $\lceil i_D \rceil$, grows without bound as $a_0$ approaches the cutoff $a^{\prime }_0$ in (36) and becomes infinite (does not exist) when $a_0 \ge a^{\prime }_0$. The second and third cases occur under the condition $bc>d^2$, when $i_C$ may exist. Here, if $a_0$ is close to 0, then $\lceil i_D \rceil = \lfloor i_C \rfloor =1$, so $S_1$ is the only locally stable strategy for small $a_0$. When the chance of surviving alone is very small, cooperation will be advantageous except in the final step of the game. As $a_0$ increases, both $i_C$ and $i_D$ increase without bound, but with different consequences depending on whether $a^{\prime \prime }_0 < a^{\prime }_0$ or $a^{\prime \prime }_0 > a^{\prime }_0$.

The latter two cases differ owing to the different rates of increase of the two boundaries $\lceil i_D \rceil$ and $\lfloor i_C \rfloor$ as $a_0$ increases. For simplicity, we focus on the continuous interval $[i_D ,i_C]$ which has length $i_C - i_D$. We again treat *n* implicitly, knowing the picture will look different for $n<i_D$, $i_D<n<i_C$ and $n>i_C$. If $a^{\prime \prime }_0 < a^{\prime }_0$, then $i_C$ diverges before $i_D$ and $i_C - i_D$ will increase as $a_0$ increases. If $a^{\prime \prime }_0 > a^{\prime }_0$, then $i_D$ diverges before $i_C$ and $i_C - i_D$ will decrease as $a_0$ increases. In the case of shrinking $i_C - i_D$, since $\lceil i_D \rceil = \lfloor i_C \rfloor =1$ when $a_0$ is close to 0 there will be at most one locally stable state, which will exist over values of $a_0$ for which $[i_D ,i_C]$ contains an integer. Local stability becomes impossible when $a_0$ is large enough that $\lceil i_D \rceil$ exceeds $\lfloor i_C \rfloor$.

**Fig. 5:**
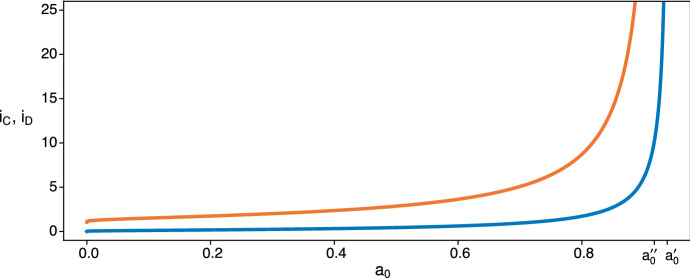
In orange, $i_C$ for a given $a_0$ is the point above which an additional round of cooperation is favored. In blue, $i_D$ for a given $a_0$ is the point below which an additional round of defection is favored. The game parameters are $a=0.97$, $b=0.93$, $c=0.98$, $d=0.95$, which are related to those used previous, e.g. in Fig. 4, by subtracting 0.01 from *b* and *c* which makes $a_0^{\prime \prime } < a_0^\prime$ while keeping $bc>d^2$. For any given $a_0$, the stretch of locally stable states is the set of integer values of *i* falling between the two lines, where increased cooperation and increased defection are both disfavored

Figure 5 illustrates the case where $a^{\prime \prime }_0 < a^{\prime }_0$, so that $i_C$ diverges before $i_D$. In Appendix 3, we prove that the stretch of equilibria grows with $a_0$ in this case. The stretch of locally stable equilibria $\llbracket \lceil i_D \rceil ,\lfloor i_C \rfloor \rrbracket$ increases in length with its two boundaries drifting towards *n* as $a_0$ grows. The upper limit $\lfloor i_C \rfloor$ will reach *n* for some $a_0 <a^{\prime \prime }_0$ after which the stretch of equilibria will be $\llbracket \lceil i_D \rceil , n \rrbracket$ which starts closing as the lower boundary increases with $a_0$. Eventually the stretch will be reduced to the single point *n* for some $a_0<a^{\prime }_0$. The stretch will disappear as $a_0$ approaches $a^{\prime }_0$, meaning that there will always be an incentive to defect once more. But since there are only *n* rounds in the game, $S_n$ will remain a stable strategy for all larger values of $a_0$.

**Fig. 6:**
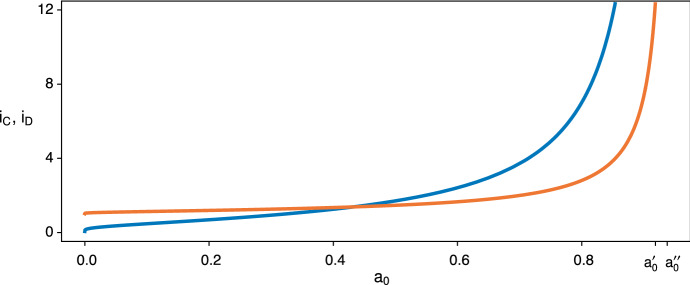
As in Fig. 5, for a given $a_0$, $i_C$ (orange) is the point above which an additional round of cooperation is favored and $i_D$ (blue) is the point below which an additional round of defection is favored. The vertical stretch between $i_D$ and $i_C$ shrinks as $a_0$ increases from 0. After the curves cross, at $a_0 \approx 0.43$ using (45) in this case, the vertical span between $i_C$ and $i_D$ is the interval where both cooperating more and defecting more are better than keeping one’s strategy. The curve for $i_D$ above is identical to the one plotted in Fig. 4 because the same parameters are used here: $a=0.97$, $b=0.94$, $c=0.99$, $d=0.95$

Using the same techniques, the opposite behavior can be shown to hold when $a^{\prime }_0 < a^{\prime \prime }_0$. Here, the stretch decreases in length, with at most one locally stable state, until it disappears at some $a_0 < a^{\prime }_0$ when the curves for $i_C$ and $i_D$ cross. Figure 6 shows an example. For $a_0$ larger than the value for which $i_C=i_D$, no stretch of locally stable equilibria can exist. We might call this value $a_0^{\prime \prime \prime }$ and for reference give its formula,

45 $$\begin{aligned} a_0^{\prime \prime \prime } = \frac{b d (c - d) (a^2 - b c)}{(a^2 - b c + c - a) (b c - d^2)} , \end{aligned}$$

which can be obtained using (37) and (42). For the parameters in Fig. 6, we have $a_0^{\prime \prime \prime } \approx 0.43$. As long as *n* is large enough, there will be three zones: for small *i* there will only be an incentive to defect more, for intermediate *i* increased defection and increased cooperation will both be favored over keeping the same strategy, and for large *i* there will only be an incentive to cooperate more. These three zones will drift towards larger *i* so that eventually for some $a_0<a^{\prime \prime }_0$ there will only be an advantage to defect one more time. Then only $S_n$ will remain a stable strategy.

#### Summary and interpretation of cases

Our analyses in the previous two sections (4.2.1 and 4.2.2) establish that when neither $i_D$ nor $i_C$ exists, there is an incentive to defect one more time against a partner with strategy $S_i$ regardless of *i*. When $i_D$ exists, additional defections are favored if $i < i_D$ but disfavored if $i > i_D$. When $i_C$ exists, additional cooperations are disfavored if $i < i_C$ but favored if $i > i_C$. We focused on the possibility of a non-empty stretch of local equilibria $\llbracket \lceil i_D \rceil ,\lfloor i_C \rfloor \rrbracket$ existing when $i>i_D$ and $i < i_C$. We also described the possibility of a stretch of what we may call ‘disequilibria’, where increased defection and increased cooperation are both favored. Here we point out a third case, that the stretch contains no integers, that is when $\lfloor i_D \rfloor = \lfloor i_C \rfloor$ so incentives switch between $\lfloor i_D \rfloor$ and $\lceil i_D \rceil$. In all scenarios, we established that when *i* is outside the stretch there is incentive to move toward it by increasing the number of defections if $i<i_D$ and increasing the number of cooperations if $i>i_C$.

Table 2 delineates ten possibilities, showing the parameter ranges and resulting incentive structures for each, assuming *n* is large enough that $n \ge \lceil i_D \rceil$ and $n \ge \lceil i_C \rceil$ whenever $i_D$ and $i_C$ exist. The first level of classification creates three major divisions: in the first, only $i_D$ may exist, whereas in the second and third both $i_D$ and $i_C$ may exist. The second, finer level emphasizes the importance of $a_0$. Among the ten possibilities listed, we recognize five basic types of incentive structure.

Roughly speaking, large $a_0$ causes additional defection to be favored regardless of *i* (Type 1), and small $a_0$ leads to the existence of equilibria which may be unbounded and capped only by *n* (Type 2) or bounded by $\lfloor i_C \rfloor$ (Type 3). For some intermediate values of $a_0$, disequilibria arise which may be unbounded (Type 4) or bounded (Type 5). These intermediate values of $a_0$ occur when $bc>d^2$ and $a_0^{\prime } < a_0^{\prime \prime }$, as in Fig. 6, and $a_0$ is larger than the value for which $i_D$ and $i_C$ cross, namely $a_0^{\prime \prime \prime }$ in (45). However, we do not use $a_0^{\prime \prime \prime }$ to classify incentive structures in Table 2 because incentive structures depend on $\lceil i_D \rceil$ and $\lfloor i_C \rfloor$, not simply on $i_D$ and $i_C$.

**Table 2:** Parameter regions—largely determined by the relative magnitude of the loner survival probability $a_0$—which produce different incentives for an individual with strategy $S_i$ to either cooperate once more, defect once more, either or neither, against a partner with the same strategy $S_i$

$$bc \le d^2$$		
$a_0 \ge a_0^{\prime }$	Additional defection always favored	(1)
$a_0 < a_0^{\prime }$	Possible stretch of equilibria $\llbracket \lceil i_D \rceil ,n \rrbracket$	(2)
$bc > d^2$ and $a_0^{\prime } \ge a_0^{\prime \prime }$		
$a_0 \ge a_0^{\prime }$	Additional defection always favored	(1)
$a_0^{\prime \prime } \le a_0 < a_0^{\prime }$	Possible stretch of equilibria $\llbracket \lceil i_D \rceil ,n \rrbracket$	(2)
$a_0 < a_0^{\prime \prime }$	Possible stretch of equilibria $\llbracket \lceil i_D \rceil ,\lfloor i_C \rfloor \rrbracket$	(3a)
$bc > d^2$ and $a_0^{\prime } < a_0^{\prime \prime }$		
$a_0 \ge a_0^{\prime \prime }$	Additional defection always favored	(1)
$a_0^{\prime } \le a_0 < a_0^{\prime \prime }$	Possible stretch of disequilibria $\llbracket \lceil i_C \rceil ,n \rrbracket$	(4)
$a_0 < a_0^{\prime }$ and $\lfloor i_D \rfloor > \lfloor i_C \rfloor$	Possible stretch of disequilibria $\llbracket \lceil i_C \rceil ,\lfloor i_D \rfloor \rrbracket$	(5a)
$a_0 < a_0^{\prime }$ and $\lfloor i_D \rfloor = \lfloor i_C \rfloor$	Incentives switch between $\lfloor i_D \rfloor$ and $\lceil i_D \rceil$	(5b)
$a_0 < a_0^{\prime }$ and $\lfloor i_D \rfloor < \lfloor i_C \rfloor$	Single equilibrium point $\lceil i_D \rceil =\lfloor i_C \rfloor$	(3b)

In all cases it is assumed that $c> a> d > b$ and $a^2 > bc$. In the second-to-last line, the incentives switch from favoring additional defection if $i \le \lfloor i_D \rfloor$ to favoring additional cooperation if $i \ge \lceil i_D \rceil = \lceil i_C \rceil$. The right-most column bins the ten possible incentive structures into the five basic types

Following the discussion of Fig. 1 in Sect. 2, we interpret the possibilities outlined in Table 2 as a balance between individual survival and pair survival. The first major division of Table 2 has already been discussed. It is based on the assumption that the order of eigenvalues is $a^2 > d^2 \ge bc$, with $a_0$ falling somewhere between 0 and 1. Here an additional round of cooperation does not benefit the individual ($b-d<0$) or the pair ($bc-d^2 \le 0$). Thus the only criterion for stable states is whether additional defections remain favored. They are favored for small *i* but become disfavored at some larger value of $i = \lceil i_D \rceil$ which increases with $a_0$. For $a_0 \ge a^{\prime }_0$, additional defections are favored for all *i* so none of the $S_i$ are stable.

The second and third major divisions of Table 2 are for $a^2> bc > d^2$, in which case the interval of locally stable states is bounded for small $a_0$ then shifts toward larger integers as $a_0$ increases. As it shifts, both ends of the continuous interval $[i_D,i_C]$ grow smoothly with $a_0$ while its width $i_C-i_D$ either expands or shrinks depending on whether $a^{\prime }_0 > a^{\prime \prime }_0$, so that $i_C$ diverges first as in Fig. 5, or $a^{\prime }_0 < a^{\prime \prime }_0$, so that $i_D$ diverges first as in Fig. 6. In the latter case, as $a_0$ increases there may be a series of single equilibrium points (Type 3b) with $\lceil i_D \rceil =\lfloor i_C \rfloor = 1, 2, 3 \ldots$, which are separated by short intervals of $a_0$ for which incentives switch between $\lfloor i_D \rfloor$ and $\lceil i_D \rceil$ (Type 5b) before the final one of these intervals occurs around $a_0^{\prime \prime \prime }$, then is followed by a series of expanding bounded stretches of disequilibria (Type 5a) as $a_0$ approaches $a^{\prime }_0$. In the simple example of Fig. 6, $i=1$ is the single equilibrium point for $a_0 \in (0,0.323)$, incentives switch between $i=1$ and $i=2$ for $a_0 \in (0.323,0.547)$, and there are expanding stretches of disequilibria for $a_0 \in (0.547,0.919=a^{\prime }_0)$.

Putting the criterion for a shrinking stretch of equilibria, and consequently the chance for disequilibria, in terms of individual versus pair survival, we have

46 $$\begin{aligned} a^{\prime }_0< a^{\prime \prime }_0 \Leftrightarrow \frac{a^2 - bc}{c-a}<\frac{bc-d^2}{d-b} . \end{aligned}$$

On the one hand, the advantages of increased defection extend to smaller $a_0$ when the resulting cost to pair survival ($a^2 - bc$) is low compared to the gain in individual survival ($c-a$). On the other hand, the advantages of increased cooperation extend to larger $a_0$ when the resulting gain in pair survival ($bc-d^2$) is high compared to the cost in individual survival ($d-b$). When this criterion (46) is met, there is a range of loner survivability for which the threat of having to survive alone is enough to support additional cooperation but not enough to prevent additional defection.

## Global properties of $A(S_j;S_i)$

Here we return to the payoff matrix $A(S_j;S_i)$ for all $i,j \in \llbracket 0,n \rrbracket$, given by (12) for $j \ge i$ and by (13) for $j \le i$. To recap: in Sect. 3 we fixed $i=0$ and asked whether an optimal response $j=J_{opt}$ existed, and in Sect. 4 we focused on $j=i$ and considered in detail the neighboring states where *j* and *i* differ by 1. Our findings about $J_{opt}$, $i_D$ and $i_C$ retain their importance in this section, where we study the full payoff matrix $A(S_j;S_i)$. In the subsections which follow, we investigate the global stability of locally stable strategies, show how $A(S_i;S_i)$ depends on *i*, and ascertain key features of a best-response walk on the surface $A(S_j;S_i)$. We continue to assume that the single-step game is a Prisoner’s Dilemma, so we have $c> a> d > b$ and $a^2 > bc$.

### Global versus local stability

Global stability is defined as follows:

47 $$\begin{aligned} S_i \mathrm {~is~a~global~equilibrium} \Leftrightarrow \forall j\ne i ~A(S_i;S_i)>A(S_j;S_i) . \end{aligned}$$

This, again, is in the sense of a strict Nash equilibrium. A globally stable state is obviously a locally stable one. Here we prove that the reciprocal is true.

We begin with the case of increasing cooperation. Specifically, we compare the difference in payoff of two individuals, one who cooperates *k* additional times and one who cooperates $k-1$ additional times, both having a partner with strategy $S_i$. Using (13) and simplifying, we have

48 $$\begin{aligned}&A(S_{i-k};S_i) - A(S_{i-k+1};S_i) \nonumber \\&\quad =~ a^{2(n-i)} (bc)^{k-1} d^{2(i-k)} \left[ (bc-d^2) \frac{a_0-d}{a_0-d^2} \right. \nonumber \\&\qquad \left. + \left( b-d - (bc-d^2) \frac{a_0-d}{a_0-d^2} \right) \left( \frac{a_0}{d^2} \right) ^{i-k} \right] . \end{aligned}$$

Here *k* ranges from 1 to *i*. Equation (48) is negative when $k=1$, due to local stability, and will change sign at most once as *k* increases from 1 to *i*. We need only check the endpoint, $k=i$, where we find

49 $$\begin{aligned} A(S_0;S_i) - A(S_1;S_i) = a^{2(n-i)} (bc)^{i-1} (b-d) < 0 . \end{aligned}$$

No additional number of cooperations is favorable against a locally stable strategy.

In the case of increasing defection, we compare the payoff of an individual who defects $k+1$ times to that of individual who defects *k* times, against a partner with strategy $S_i$. Here *k* ranges from 0 to $n-1$, but we must consider all $k \ge 0$ because *n* may take any value. Using (12) and simplifying, we may write this difference as

50 $$\begin{aligned}&A(S_{i+k+1};S_i) - A(S_{i+k};S_i) \nonumber \\&\quad = a^{2(n-i-k-1)} a_0^i (bc)^{k} \left[ H + (c-a+a^2-bc) \frac{a_0-a_0^*}{a_0-bc} \left( \frac{a_0}{bc} \right) ^k \right] \end{aligned}$$

in which $a_0^*$ is the cutoff given by (20), which was derived in the consideration of an optimal number of defections against a partner with strategy $S_0$, and

51 $$\begin{aligned} H = (a^2-bc) \left( \frac{c(1-b)}{a_0-bc} - \frac{d(1-d)}{a_0-d^2} - \frac{a_0-d}{a_0-d^2} \left( \frac{d^2}{a_0}\right) ^i \right) , \end{aligned}$$

which does not depend on *k*. Local stability means that (50) is negative when $k=0$. If it remains negative for all $k>0$, then no additional defections will be favored against a partner with strategy $S_i$. This will depend on the comparison of *H* and the second term inside the brackets in (50). If $a_0<bc$, this second term is positive, so from $k=0$ we know *H* must be negative. Also, the second term will shrink to zero as *k* increases because $a_0/bc<1$. Therefore, the whole of (50) remains negative for all *k* if $a_0<bc$. Alternatively, if $bc< a_0 < a_0^*$, then the second term in (50) is negative and increases in absolute value as *k* increases. Here too (50) remains negative for all *k*. We do not need to consider $a_0>a_0^*$ because local stability requires $a_0 < a_0^\prime$ and we have $a_0^\prime \le a_0^*$. If $S_i$ is locally stable, there is no increased number of defections which is better.

Taking both cases together, we have proven that locally stable states and globally stable states are the same. For brevity, we have omitted the detailed treatments of special cases, such as $a_0=a^2$, and simply note that these do not alter our conclusion. In sum, globally stable states form the same intervals as locally stable states we described previously in Sect. 4.2.2.

### The diagonal $A(S_i;S_i)$

Although potentially long stretches of local equilibria may exist, not all $A(S_i;S_i)$ are equivalent. In the single-step survival game or in the usual Prisoner’s Dilemma with $a>d$, *C* is a better choice than *D* if both players take the same strategy. Here we are interested in whether $S_0$ is the best strategy in this sense in the *n*-step game. We base our analysis on the one-step difference which upon simplification may be written

52 $$\begin{aligned} A(S_{i+1};S_{i+1})-A(S_i;S_i)= & {} a^{2(n-i-1)} a_0^i (d-a) \nonumber \\&\left[ 1 + (a+d) \frac{a_0 -d}{a_0 -d^2} \left( \left( \frac{d^2}{a_0}\right) ^i - 1 \right) \right] . \end{aligned}$$

For the smallest *i* we have

53 $$\begin{aligned} A(S_1;S_1)-A(S_0;S_0) = a^{2(n-1)} (d-a) < 0 . \end{aligned}$$

The difference $A(S_{i+1};S_{i+1})-A(S_i;S_i)$ will remain negative for larger *i* unless the second term in the brackets in (52) becomes too large in the negative direction. Of course $a+d>0$. This second term in the brackets is a decreasing function of $a_0$, which begins positive for $0<a_0<d$, then becomes negative when $a_0>d$ and continues to decrease as $a_0$ approaches 1. It is straightforward to check that even with $a_0=1$, $A(S_{i+1};S_{i+1})-A(S_i;S_i)$ in (52) is negative. Thus, $A(S_i;S_i)$ is a decreasing function of *i*. The fully cooperative strategy $S_0$ is the best if both players are restricted to having the same strategy.

### A best-response walk on the surface $A(S_j;S_i)$

To better understand the full payoff matrix $A(S_j;S_i)$ for all $i,j \in \llbracket 0,n \rrbracket$, we studied the best responses of an individual to their partner’s current strategy. We assume that both players initially have the same strategy, that the partner follows suit with the same best response as the individual, and that this process is repeated. The resulting walk is well defined in the sense that none of the $A(S_j;S_i)$ are equal, considering all $j \in \llbracket 0,n \rrbracket$ for a given *i*. Because the walk is deterministic and has a finite number of possibilities (exactly $n+1$ states), it cannot be injective. Ultimately it will end in a cycle, which might consist of one globally stable strategy. As we do not consider mixed strategies or population-frequencies of strategies, these best-response walks should not be confused with best-response dynamics the way the latter are usually defined (Hofbauer and Sigmund 1998; Cressman 2005; Sandholm 2010).

The analysis of $i_D$ and $i_C$ in Sect. 4.2, based on single-step changes in strategy, shows that there is incentive to move toward a stretch of equilibria for any partner strategies outside the stretch, by increasing defection when $i \le \lfloor i_D \rfloor$ and by increasing cooperation when $i \ge \lceil i_C \rceil$. The same analysis shows that there is incentive to move toward a stretch of disequilibria or a stretch of equilibria which is empty. Here we investigate how best-response walks on the surface $A(S_j;S_i)$ depend on the initial value of *i*, how stretches of equilibria or disequilibria are approached from above and below in steps which may be greater than one, and how these walks converge on single points or enter into larger cycles.

**Fig. 7:**
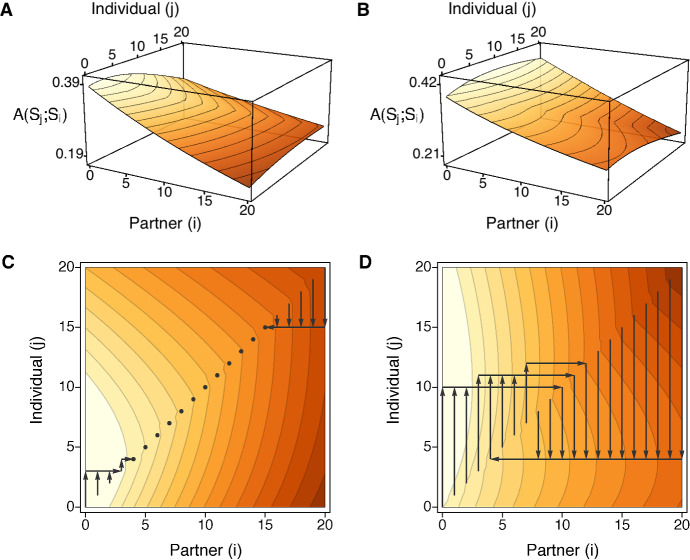
Panels **a** and **b** show two payoff surfaces, $A(S_j;S_i)$, for a game of length $n=20$. In both: $a_0=0.86$. In **a**: $(a,b,c,d)=(0.97,0.93,0.98,0.95)$ as in Fig. 5. In **b**: $(a,b,c,d)=(0.97,0.94,0.99,0.95)$ as in Fig. 6. Panels **c** and **d** show the different possible best-response walks on the same two surfaces. In **c** (and **a**), $i_D = 3.08$ and $i_C = 15.99$ and there is a stretch of equilibria for $i=4$ to $i=15$ which is approached from above and below. In **d** (and **b**) $i_C = 4.22$ and $i_D = 13.49$ and there is a stretch of disequilibria for $i=5$ to $i=13$, leading in this case to a two-state cycle between $i=4$ and $i=11$

Figure 7 illustrates this for two survival games of length $n=20$, one with a stretch of equilibria and one with a stretch of disequilibria. The first (Fig. 7a, c) has $a_0< a_0^{\prime \prime } < a_0^{\prime }$ and $0< \lceil i_D \rceil< \lfloor i_C \rfloor < n$ and so exemplifies the fifth of the ten possibilities listed in Table 2, with a stretch of equilibria for $i \in \llbracket 4,15 \rrbracket$. The second (Fig. 7b, d) has $a_0< a_0^{\prime } < a_0^{\prime \prime }$ and $0< \lceil i_C \rceil< \lfloor i_D \rfloor < n$ and so exemplifies the eighth of the ten possibilities listed in Table 2, with a stretch of disequilibria for $i \in \llbracket 5,13 \rrbracket$. Panels A and B give 3d depictions of $A(S_j;S_i)$ as a continuous surface. Panels C and D show the same surfaces, viewed from above, and display all possible best-response walks using arrows. Each possible walk starts at some point on the diagonal. It follows the vertical arrow which goes either up or down to the optimal strategy $S_j$ against $S_i$. Then it follows the horizontal arrow which goes back to the diagonal. It continues in like manner, repeating the exact same procedures.

Figure 7 shows the characteristic features of walks when $i_D$ and $i_C$ exist. In particular, if $i \le \lfloor i_D \rfloor$ the best response (on the side of additional defection) is an increasing function of *i*, whereas if $i \ge \lceil i_C \rceil$ the best response (on the side of additional cooperation) does not depend on *i*. When there is a stretch of equilibria, $\llbracket \lceil i_D \rceil ,\lfloor i_C \rfloor \rrbracket$, the points on the interior are their own best responses, and walks which begin outside the stretch converge on its endpoints, $\lceil i_D \rceil$ from below and $\lfloor i_C \rfloor$ from above. When there is a stretch of disequilibria, $\llbracket \lceil i_C \rceil ,\lfloor i_D \rfloor \rrbracket$, incentives to defect more send walks into the interior then through the stretch, toward $\lceil i_D \rceil$, but these are opposed by incentives to cooperate more, which always leap over the stretch, directly to $\lfloor i_C \rfloor$. In this case, walks may converge on cycles of two or more states.

We can use (48) and (50) in Sect. 5.1 to obtain the best responses for $i \ge \lceil i_C \rceil$ and $i \le \lfloor i_D \rfloor$, respectively. In the first case, we put $j=i-k$ in (48) and rewrite it for our purposes here as

54 $$\begin{aligned} A(S_j;S_i) - A(S_{j+1};S_i) =&~ a^{2(n-i)} (bc)^{i-j-1} d^{2j} \left[ (bc-d^2) \frac{a_0-d}{a_0-d^2} \right. \nonumber \\&\left. - (b - d + bc - d^2) \frac{a_0-a_0^{\prime \prime }}{a_0-d^2} \left( \frac{a_0}{d^2} \right) ^{j} \right] . \end{aligned}$$

Now *j* ranges from 0 to *i*. We know that (54) is negative when $j=0$, from (49) which holds for all *i*. In addition, because here we are assuming $i \ge \lceil i_C \rceil$, we know that (54) is positive when $j=i$. We treat *j* as continuous and solve for the value which makes (54) equal to zero,

55 $$\begin{aligned} j^{*} = \frac{ \ln { \left( \frac{bc-d^2}{b-d+bc-d^2}\frac{a_0-d}{a_0-a_0^{\prime \prime }} \right) } }{\ln {\left( \frac{a_0}{d^2}\right) }} . \end{aligned}$$

Then, the best response falls in the interval $(j^{*},j^{*}+1)$ and must be equal to $\lceil j^{*} \rceil$. Writing (55) in this way emphasizes that we are considering the case $a_0<a_0^{\prime \prime }$, namely when $i_C$ exists. In fact, it is straightforward to show that $j^{*} = i_C - 1$, so that $\lceil j^{*} \rceil = \lfloor i_C \rfloor$. Thus, for partner strategies with $i \ge \lceil i_C \rceil$, the optimal strategy of an individual is to defect only in the final $\lceil j^{*} \rceil = \lfloor i_C \rfloor$ steps of the game. If there is a stretch of equilibria then $\lceil j^{*} \rceil$ is at the upper end of the stretch, whereas if there is a stretch of disequilibria then $\lceil j^{*} \rceil$ is just beyond the lower end of the stretch.

In the second case, $i \le \lfloor i_D \rfloor$, we similarly set (50) equal to zero and solve to obtain

56 $$\begin{aligned} k^{*}(i) = \frac{ \ln { \left( \frac{-H(a_0-bc)}{(c-a+a^2-bc) (a_0-a_0^*)} \right) } }{\ln {\left( \frac{a_0}{bc}\right) }} \end{aligned}$$

in which the dependence on *i* is through *H*, given by (51). The best response is captured by the interval $(i+k^{*}(i),i+k^{*}(i)+1)$ and is equal to $i + \lceil k^{*}(i) \rceil$. The full expression for $k^{*}(i)$ is cumbersome, but for the smallest *i* we have

57 $$\begin{aligned} k^{*}(0) = \frac{ \ln { \left( \frac{a^2-bc}{c-a+a^2-bc}\frac{a_0-c}{a_0-a_0^{\star }} \right) } }{\ln {\left( \frac{a_0}{bc}\right) }} . \end{aligned}$$

Note that this is another route to the optimal number of defections against a fully cooperative partner (Sect. 3.2) because $\lceil k^{*}(0) \rceil = J_{opt}$. For larger *i*, we find that $k^{*}(i)$ decreases with *i*, finally reaching zero for $i=i_D$. As Fig. 7 shows, the optimal total number $(i + \lceil k^{*}(i) \rceil )$ of end-game defections against partner strategies with $i \le \lfloor i_D \rfloor$ increases with *i*. The largest integer-valued *i* which still favors increased defection is $i = \lfloor i_D \rfloor$ and this would motivate one additional defection by the individual, up to $j = \lceil i_D \rceil$. If there is a stretch of equilibria, this largest value is at the lower end of the stretch, whereas if there is a stretch of disequilibria it is just beyond the upper end of the stretch. However, in the latter case, as the walk moves through the stretch, it may happen as in Fig. 7d that it never reaches $j = \lceil i_D \rceil$ and instead turns downward because there is an even stronger incentive for additional cooperation.

**Fig. 8:**
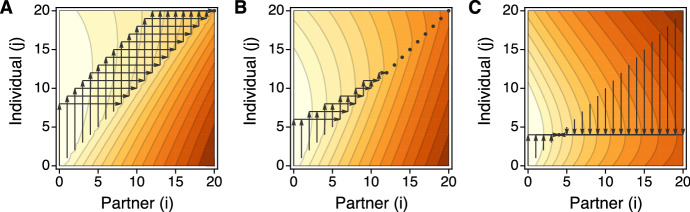
Three additional examples of best-response walks on the surface $A(S_j;S_i)$ for a game of length $n=20$. In **a**, $(a,b,c,d)=(0.97,0.91,0.98,0.95)$ and $a_0=0.95$, so $bc<d^2$ and $a_0 > a_0^\prime$, and additional defection is always favored. In **b**, $(a,b,c,d)=(0.97,0.93,0.98,0.95)$ and $a_0=0.92$, so $bc>d^2$ and $a_0^{\prime \prime }< a_0 < a_0^\prime$, and all $i \ge 12$ are stable. In **c**, $(a,b,c,d)=(0.97,0.93,0.99,0.949)$ and $a_0=0.78$, so $bc>d^2$, $a_0< a_0^{\prime } < a_0^{\prime \prime }$ and $\lfloor i_D \rfloor < \lfloor i_C \rfloor$, and there is a single stable state at $i = 4$. Thus, these correspond to the first, third and last of the ten possibilities listed in Table 2

The examples in Fig. 7 represent just two of the five distinct outcomes among the ten total possibilities listed in Table 2, namely when there is either a bounded stretch of equilibria (Type 3a) or a bounded stretch of disequilibria (Type 5a), and *n* is large enough that the entire stretch is apparent within the game. Figure 8 shows three more outcomes: a case in which additional defection is favored for all *i* (Fig. 8a), a case in which there is a stretch of equilibria capped by *n* (Fig. 8b), and a case in which there is a single equilibrium point (Fig. 8c). These are the first (Type 1), fourth (Type 2), and tenth (Type 3b) of ten possibilities in Table 2. The ninth possibility in Table 2 (Type 5b), when incentives switch between $\lfloor i_D \rfloor = \lfloor i_C \rfloor$ and $\lceil i_D \rceil = \lceil i_C \rceil$, is not depicted but will simply result in a cycle between those two adjacent states. We have also not depicted an example of the seventh possibility (Type 4) but note that it produces multi-state cycles similar to what is shown in Fig. 7b, d.

We may also consider $k^{*}(i)$ and $j^*$ separately for $j \ge i$ above the diagonal and $j \le i$ below the diagonal. Best responses on the side of increasing defection ($j \ge i$) are tentative in the sense that they proceed incrementally, the optimum for a given *i* being $j=i+\lceil k^{*}(i) \rceil$. When the upper limit to increasing defection $i_D$ exists, the best-response walk leads to it, but generally in increments of decreasing size as in Fig. 8b rather than all at once. When $i_D$ does not exist, a richer set of possibilities can occur, depending on the value of $a_0$. If $a_0<d$, then $\lceil k^{*}(i) \rceil$ still decreases with *i*, from $\lceil k^{*}(0) \rceil = J_{opt}$ down to its lower limit of $\lceil k^{*}(i) \rceil = 1$ for some larger *i*. If $a_0=d$, then $\lceil k^{*}(i) \rceil$ is a constant ($J_{opt}$) as in Fig. 8a. If $a_0>d$, then $\lceil k^{*}(i) \rceil$ is an increasing function of *i*. We prove these statements in Appendix 4. In sum, the best-response number of defections, $j=i+\lceil k^{*}(i) \rceil$, is an increasing function of *i*, approaching $i_D$ in steps if $i_D$ exists and otherwise stopping only when it hits the cap *n*. Note (cf. Fig. 8c) that we use increasing to mean non-decreasing, as opposed to strictly increasing.

In contrast, the result $j^*$ says that when $i_C$ exists, then for any partner strategies with $i \ge \lceil i_C \rceil$, the best-response number of defections is $\lfloor i_C \rfloor$. There is no incentive to try some intermediate number of defections but only to jump straight to the endpoint of the best-response walk on the side of increasing cooperation. Of course, $i_C$ might not exist and then there is never an incentive to cooperate more. But when $i_C$ does exist, the best option is to cooperate from the beginning of the game and to continue cooperating for $n-\lfloor i_C \rfloor$ steps, even if the partner is going to defect in every round. Note that a decision to cooperate more comes from a position of both players already defecting too much ($i>i_C$), so the prospect and resulting cost of having to survive alone are high. Additional cooperation by the individual also directly benefits the partner, which is different than the case of increasing defection when $i<i_D$, where the interests of the individual and the partner are not aligned.

## The parameter space of Prisoner-Dilemma survival games

Throughout this work, we have been particularly interested in whether cooperation can become favored upon iteration, due to low loner survivability, despite a single-step Prisoner’s Dilemma. In this section, we characterize the parameter space of single-step games to see how broadly this holds. We sample parameter sets randomly, subject to (17) and (18), i.e. $c> a> d > b$ and $a^2 > bc$, then bin them according to the five possible incentive structures introduced in Table 2 and explored in terms of best-response walks in Sect. 5. Here, we describe these five qualitatively different incentive structures simply as in Table 3. We may say that cooperation is supported, in the sense of there being at least some checks on defection, under four of the five incentive structures (Types 2-5) but is clearly not supported when additional defection is always favored regardless of the partner’s strategy (Type 1).

Table 3 gives the results when the survival probabilities ($a,b,c,d,a_0$) are sampled uniformly at random under two different models. The first model draws from the entire parameter space. This admits many cases where having a partner at all is an obvious disadvantage ($a_0 > a,b,c,d$) and where the prospects of surviving even a single round may be low. The second model samples over two narrower ranges: (0.9, 1) for *a*, *b*, *c* and *d*, and (0.7, 1) for $a_0$. This focuses on games which are not too harsh in a single step, and in which $a_0$ may be relatively small but may still exceed *a*, *b*, *c*, and *d*. Note that the cutoffs for cooperation possibly being favored fall between pairwise and individual survival probabilities: $a^2< a_0^*< a$ and $d^2 < a_0^\prime$, $a_0^{\prime \prime } < d$. In this second model, the lower bound for all pairwise survival probabilities is $0.9^2 = 0.81$, so there is a strong chance a loner will be worse off than a pair of individuals.

**Table 3:** Percent outcomes for one million parameter sets sampled uniformly at random according to two different models, in which the single-step game is a Prisoner’s Dilemma ($c>a>d>b$ and $a^2>bc$)

Incentive structure	Models for random sampling	
	$a,b,c,d \in (0,1)$	$a,b,c,d \in (0.9,1)$
	$a_0 \in (0,1)$	$a_0 \in (0.7,1)$
1. Defection always favored	66.59	22.95
2. Unbounded stretch of equilibria	24.22	30.20
3. Bounded stretch of equilibria	6.70	25.69
4. Unbounded stretch of disequilibria	1.04	2.31
5. Bounded stretch of disequilibria	1.45	18.85

The descriptions of incentive structures 2 through 5 hold when the cap *n* is large enough ($n>i_D, i_C$)

We generated one million random samples of parameters for each model. For each sample, we took four uniform random numbers in the appropriate range, sorted and labeled them so that $c>a>d>b$. Thus, all one million initial samples satisfied assumption (17). We then excluded samples which did not meet assumption (18), namely $a^2 > bc$. This excluded about 10% of samples in the first model and about 24% in the second model. For each remaining sample, we generated an $a_0$ uniformly at random, again in the range for each model. Finally, we checked the samples against the criteria in Table 2, binned them into the five qualitatively different incentive structures, and computed the percentages of samples falling into each bin.

Under the first model, across the entire parameter space of these games, additional defection is favored against any partner strategy about two-thirds of the time. Most of the other one-third is occupied by games with unbounded stretches of equilibria. Games with stretches of disequilibria are rare. Under the second model, with a restricted parameter space which should tend to favor cooperation, defection is favored regardless of partner strategy less than one-quarter of the time. Bounded stretches of equilibria or disequilibria are more frequent. Unbounded stretches of disequilibria remain rare, which makes sense as this requires $a_0$ to fall between $a_0^\prime$ and $a_0^{\prime \prime }$.

We may also quantify the extent of checks on the number of end-game defections in cases where cooperation is supported. Due to the shapes of $i_D$ and $i_C$ as functions of $a_0$ (recall Figs. 5 and 6), which remain relatively flat until diverging sharply as $a_0$ approaches $a_0^\prime$ and $a_0^{\prime \prime }$, there are essentially two kinds of games. On the one hand, if $a_0 \ge a_0^\prime ,a_0^{\prime \prime }$, then defection is clearly favored. On the other hand, if $a_0<a_0^\prime$ or $a_0<a_0^{\prime \prime }$, then there are rather strong checks on defection. Across all cases in which $i_D$ or $i_C$ existed under the first model in Table 3, the median $i_D$ was 0.3 and the 90th percentile $i_D$ was 2.0. The median $i_C$ was 1.6 and the 90th percentile $i_C$ was 4.6. In the second model, the median $i_D$ was 2.4 and the 90th percentile $i_D$ was 16.8; the median $i_C$ was 4.5 and the 90th percentile $i_D$ was 22.5.

## Discussion

We studied the effects of switching from *C* to *D* at some point during an iterated, two-player survival game. We focused on the case where each single step is a canonical Prisoner’s Dilemma ($c> a> d > b$, $a^2 > bc$) and asked how the temptation to defect on one’s partner might be offset by the threat of having to survive alone. We found three critical values for the loner survival probability ($a_0^*,a_0^\prime ,a_0^{\prime \prime }$) which establish broad patterns of incentives to cooperate more or defect more. When $a_0$ is small relative to these values, choosing to defect early in the game is disadvantageous and more cooperative strategies are supported. The opposite is true when $a_0$ is large.

Our model of strategy choice in an iterated survival game complements previous ones which have assumed that individuals possess fixed single-step strategies. Eshel and Weinshall (1988) modeled single-step strategies as probabilistic mixtures of *C* and *D*, with *n* geometrically distributed and (*a*, *b*, *c*, *d*) drawn from a distribution with non-zero probabilities for Harmony Games $(a \ge c,b \ge d)$ as well as Prisoner’s Dilemmas $(c>a>d>b)$. Eshel and Shaked (2001) added the possibility of non-independence of players’ survival within each step. Garay (2009) considered mixtures like those of Eshel and Weinshall (1988) but in a game of fixed length and with constant single-step payoffs. Wakeley and Nowak (2019) studied the choice between the two pure, single-step strategies, *C* and *D*, in games of fixed length.

We constrained our players to choose among the pure *n*-step strategies, $S_i$ in which *i* is the number of end-game defections. This limits their options from $2^n$ possible *n*-step strategies to an array of $n+1$ strategies. Their only question is when to start defecting. They decide this before the game begins, rather than reactively during the game. Also, though they may end up alone, the only way for this to happen is for their partner to die. When $a_0$ is large, our model suggests defecting early in hopes of becoming a loner soon. Throughout this work, we have been motivated by the other possibility, that individuals might want to stay in the game to avoid becoming a loner when $a_0$ is not too large, in which case our model is a lot more palatable. But in either scenario, it would be of interest to loosen our restrictions and allow individuals to react and to become loners without causing their partner’s demise.

Outside the context of iterated survival games, two lines of related work have shown how cooperation can be supported when individuals may opt out of interactions. In the first approach, individuals may decide to opt out of a game when loners receive a separate, potentially viable payoff (Hauert et al. 2002a, b; Garcia et al. 2015; Rossine et al. 2020). In our model, a decision to terminate a partnership would result in both players becoming loners, each surviving by $a_0$ for the remaining steps of the game. Depending on how this was implemented, opting out could become more attractive than defecting (and waiting for the partner to die) when $a_0$ is large.

In the second approach, individuals may opt out depending on what the partner has done—especially if the partner defects—and then obtain a new partner (Izquierdo et al. 2010, 2014; Zhang et al. 2016; Zheng et al. 2017). This provides a mechanism for positive assortment in interactions, which is known generally to support cooperation (Eshel and Cavalli-Sforza 1982; Taylor and Nowak 2006). In our model, rejecting one partner and getting another at some step of the game seems at odds with the basic principle that loner survivability should matter. But if assortment took the form of propensities, so that the payoff for $S_j$ was an average over partner strategies $S_i$ which favored $i=j$, we might infer from Sect. 5.2 that additional defection would be even less advantageous for small $a_0$ than we find here.

A two-player iterated survival game is a game against Nature in which individuals may succeed by working together. Our players can do this even when choosing strategies which maximize individual survival at the possible expense of the partner. But their success is limited. We have shown that stretches of equilibria may exist (Table 2, Type 2 and 3) where individuals have no incentive to change strategy. We have also shown (Sect. 5.2) that both players simultaneously cooperating one more time is advantageous regardless of their current strategy. The fact that best-response walks get stuck at $i = \lfloor i_C \rfloor$ when they start with $i > \lfloor i_C \rfloor$ and never move if they begin inside a stretch of equilibria $(\lceil i_D \rceil< i < \lfloor i_C \rfloor )$ illustrates the shortcomings of the rational, myopic, noncooperative players of traditional game theory (Binmore 1987).

One way in which players could access better equilibria would if they were able to collaborate (Sugden 1993; Bacharach 1999; Newton 2017; Rusch 2019). They might, for example, make a rational agreement to consider joint changes in strategy which benefitted both if no unilateral change was advantageous. If such players were constrained to choose among adjacent strategies as in Sect. 4, this would allow them to proceed through potentially long stretches of equilibria, increasing cooperation until they reached $i = \lceil i_D \rceil$. They would then cycle between $\lceil i_D \rceil - 1$ and $\lceil i_D \rceil$. If instead such players could choose among all strategies as in Sect. 5, they would jump from any equilibrium strategy straight to the best pairwise strategy, at $i=0$, where they would then see the advantageous unilateral move to $J_{opt}$. The end result would be a cycle including $i = \lceil i_D \rceil$ and $i=0$ (cf. Fig. 7c and Fig. 8b, c).

Another way to allow the exploration of more favorable equilibria would be for strategy selection to occur via evolution in a finite population (Young and Foster 1991; Kandori et al. 1993; Binmore et al. 1995; Amir and Berninghaus 1996; Binmore and Samuelson 1997; Nowak et al. 2004; Fudenberg et al. 2006; Sandholm 2010). This could be done by including multiple strategies and mutations in the finite population model of Wakeley and Nowak (2019). Although the details would depend on the size and structure of the population as well as on the structure of mutation—e.g. nearest-neighbor as in Sect. 4 or distributed in some way over all possible strategies—we expect that the attainment of mutually beneficial states like $i = \lceil i_D \rceil$ would be facilitated relative to our best-response walk or to the infinite-population replicator dynamic (Taylor and Jonker 1978).

This extension to finite populations would not be as simple as the Moran model (Moran 1958, 1962) of Binmore et al. (1995) because both individuals may die in our survival game, but we expect similar results would hold. At stationarity with high mutation rates, multiple strategies would be present in the population at once, whereas with low mutation rates, the population would be monomorphic most of the time. In both cases, the full range of equilibrium strategies could be explored, and in the latter case the population would be concentrated on particular ones of these (Binmore et al. 1995; Fudenberg et al. 2006; Sandholm 2007, 2010). We might predict that individuals’ strategies would vary around $\lceil i_D \rceil$ when stretches of equilibria exist. We might also speculate that when stretches of disequilibria exist, so that $\lceil i_C \rceil$ is the best state in the stretch when both players have the same strategy, finite-population dynamics could offset the tendency to cycle back to less mutually favorable states.

Our model of switching strategies in a two-player iterated survival game essentially provides a mechanism for ‘by-product mutualism’ (West-Eberhard 1975; Brown 1983; Mesterton-Gibbons and Dugatkin 1992) specifically where cooperative behaviors are supported if the longer-term consequences of cooperation versus defection are taken into account (Mesterton-Gibbons and Dugatkin 1997; Garay 2009; Smaldino et al. 2013; De Jaegher and Hoyer 2016). We have shown how iteration can change the game so as to favor cooperation (De Jaegher 2019), notably without any details of behavior or ecology, only the multiplicative accrual of survival payoffs.

## Figures and Tables

**Fig. 9:**
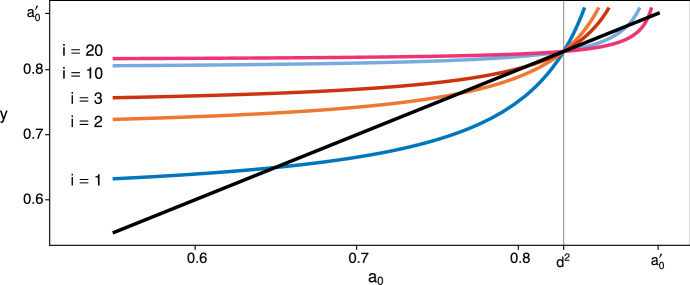
Solving (63) graphically means finding the intersection between the diagonal $y=a_0$ in black and one of the curves in color $y=d^{2}\left( 1+ \frac{c-a}{a^2 -bc}\frac{a_0 -d^2}{a_0 -d} \right) ^{-1/i}$ for a given *i*. This is illustrated here for five different values of *i* and game parameters $a=0.97$ , $b=0.9$ , $c=0.99$ and $d=0.91$. These differ from the parameters in Fig. 4 and previous figures by subtracting 0.04 from *b* and *d*, which makes $d^2$ smaller while keeping $a_0^\prime$ close to *d*, in order to illustrate the curves in the region $a_0>d^2$

**Fig. 10:**
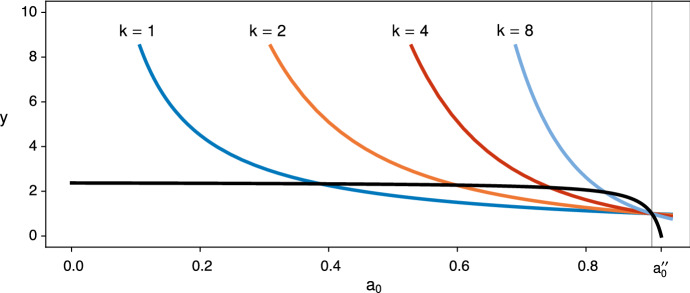
Solving (66) graphically means finding the intersections of the colored curves $y=\left( \frac{d^2}{a_0} \right) ^k$ for different values of *k* and the single black curve $y=\left( 1 + \frac{d-b}{bc-d^2} \frac{a_0 -d^2}{a_0 -d}\right) \left( 1 +\frac{c-a}{a^2 - bc} \frac{a_0 -d^2}{a_0 -d}\right) ^{-1}$. The parameters are $a=0.97$, $b=0.93$, $c=0.98$, $d=0.95$ as in Fig. 5. All curves intersect when $a_0=d^2$, which is close to $a^{\prime \prime }_0$ in this case and marked by the thin vertical line

## Appendix 1

To prove that $J_{opt}$ increases with $a_0$ for $a_0 < a^{*}_0$, we note that

58 $$\begin{aligned} J_{opt} = 1 + max \left\{ j \ge 1 \vert A(S_{j+1};S_0) \ge A(S_j;S_0) \right\} . \end{aligned}$$

Therefore, it is enough to prove that, for any given *j*, if $A(S_{j+1};S_0) \ge A(S_j;S_0)$ for some $a_0$ then it is also true for any larger $a_0$. In the special case $j=0$, we have $A(S_1;S_0) \ge A(S_0;S_0)$ for all $a_0$ because in this case (19) does not depend on $a_0$. Let *j* be some integer such that $n>j \ge 1$. Then we have

59 $$\begin{aligned}&(A(S_{j+1};S_0) - A(S_j;S_0))(a_0) = a^{2(n-j-1)} \left( c-a + a^2 -bc \right) a^{j}_0 \nonumber \\&\qquad - a^{2(n-j-1)} (a^2 -bc) (bc)^{j} - a^{2(n-j-1)}(a^2 -bc)c(1-b) \sum _{k=0}^{j-1} (bc)^k a^{j-k-1}_{0} \end{aligned}$$

60 $$\begin{aligned}&\frac{\mathrm {d~~}}{\mathrm {d} a_0}(A(S_{j+1};S_0) - A(S_j;S_0))(a_0) = ja^{2(n-j-1)} \left( c-a + a^2 -bc \right) a^{j-1}_0 \nonumber \\&\qquad - a^{2(n-j-1)}(a^2 -bc)c(1-b) \sum _{k=0}^{j-2} (j-k-1)(bc)^k a^{j-k-2}_{0} \end{aligned}$$

61 $$\begin{aligned}&\ge \frac{j}{a_0} \Bigg [a^{2(n-j-1)} \left( c-a + a^2 -bc \right) a^{j}_0\nonumber \\&\qquad - a^{2(n-j-1)}(a^2 -bc)c(1-b) \sum _{k=0}^{j-2} (bc)^k a^{j-k-1}_{0} \Bigg ] \end{aligned}$$

62 $$\begin{aligned}&\ge \frac{j}{a_0} (A(S_{j+1};S_0)- A(S_j;S_0))(a_0) + \frac{j}{a_0}a^{2(n-j-1)} (a^2 -bc) (bc)^{j} . \end{aligned}$$

Then since $a^2 > bc$, the last inequality completes the proof. When $A(S_{j+1};S_0)- A(S_j;S_0)\ge 0$ for some $a_0$ it remains positive for any larger $a_0$. With an all-*C* partner, $J_{opt}$ increases with $a_0 < a^{*}_0$.

## Appendix 2

To prove that $i_D$ is an increasing function of $a_0$, we focus on the point at which defecting one more time switches from being advantageous to being disadvantageous. This determines the relationship between $i_D$ and $a_0$, namely

63 $$\begin{aligned}&A(S_{i+1};S_i) - A(S_i;S_i) = 0 \nonumber \\&\qquad \Leftrightarrow (bc-a^2) \frac{a_0 -d}{a_0 -d^2} d^{2i} +\left( (a^2 -bc) \frac{a_0 -d}{a_0 -d^2} +c-a\right) a^{i}_0 =0 \nonumber \\&\qquad \Leftrightarrow \frac{d^{2}}{\left( 1+ \frac{c-a}{a^2 -bc}\frac{a_0 -d^2}{a_0 -d} \right) ^{\frac{1}{i}}}=a_0 . \end{aligned}$$

Both $i = i_D$ and $a_0 = d^2$ are solutions of (63). The solution $a_0 = d^2$ is true for all *i*. We want to know how the other solution depends on $a_0$, and for this we write $i_D(a_0)$. We use a graphical method depicted in Fig. 9. Specifically, the two solutions of (63) are the two points at which the diagonal $y=a_0$ and the curve $y=d^{2}\left( 1+ \frac{c-a}{a^2 -bc}\frac{a_0 -d^2}{a_0 -d} \right) ^{-1/i}$ intersect for a given *i*. Every one of these curves crosses the diagonal at $a_0=d^2$. The other point of intersection depends on *i* and, for each curve, happens at $a_0$ such that $i_D (a_0)$ solves (63). Under the assumptions (17) and (18), the function $d^{2}\left( 1+ \frac{c-a}{a^2 -bc}\frac{a_0 -d^2}{a_0 -d} \right) ^{-1/i}$ increases with $a_0$ and, for a given $a_0<d^2$, it increases with *i*. Then because these curves are anchored at $a_0=d^2$, the other points at which they cross the diagonal, which we call $a_{0}(i)$, must also increase with *i*. Considering two values of *i*, with $i_1> i_2 > 0$, we have

64 $$\begin{aligned} a_0 < d^2 \Rightarrow \frac{d^{2}}{\left( 1+ \frac{c-a}{a^2 -bc}\frac{a_0 -d^2}{a_0 -d} \right) ^{\frac{1}{i_1}}} > \frac{d^{2}}{\left( 1+ \frac{c-a}{a^2 -bc}\frac{a_0 -d^2}{a_0 -d} \right) ^{\frac{1}{i_2}}} \end{aligned}$$

so that $a_{0}(i_2)<d^2 \Rightarrow a_{0}(i_1)>a_{0} (i_2)~ \mathrm { and }~ a_{0}(i_2)>d^2 \Rightarrow a_{0}(i_1)>d^2$, and

65 $$\begin{aligned} a_0 > d^2 \Rightarrow \frac{d^{2}}{\left( 1+ \frac{c-a}{a^2 -bc}\frac{a_0 -d^2}{a_0 -d} \right) ^{\frac{1}{i_1}}} <\frac{d^{2}}{\left( 1+ \frac{c-a}{a^2 -bc}\frac{a_0 -d^2}{a_0 -d} \right) ^{\frac{1}{i_2}}} \end{aligned}$$

so that $a_{0}(i_2)>d^2 \Rightarrow a_{0}(i_1) >a_{0}(i_2)$. Finally, because $a_0 (i)$ is a positive strictly increasing function, its reciprocal function $i_D (a_0)$ is a strictly increasing function, which is what we set out to prove.

## Appendix 3

A graphical proof shows that the stretch of equilibria grows with $a_0$ in the case $a^{\prime \prime }_0 < a^{\prime }_0$. Assume some $k>0$. Then we have

66 $$\begin{aligned} i_C = i_D + k+1 \Leftrightarrow \left( \frac{d^2}{a_0} \right) ^k = \frac{1 + \frac{d-b}{bc-d^2} \frac{a_0 -d^2}{a_0 -d}}{1 +\frac{c-a}{a^2 - bc} \frac{a_0 -d^2}{a_0 -d}} . \end{aligned}$$

As shown in Fig. 10, graphing the two sides of the right-hand equality in (66) as functions of $a_0$ shows that the two curves intersect at $a_0=d^2$ regardless of *k*. This point anchors all the curves, though it is not a permissible solution of (66) because (66) was derived assuming $d^2 \ne a_0$. For any given *k*, the two curves intersect again at another $a_0$ which is the solution of (66) and which increases with *k*. We call this value $a_{0}(k)$. Then for $k_1>k_2 >0$,

67 $$\begin{aligned} a_0 \le d^2 \Rightarrow \left( \frac{d^2}{a_0} \right) ^{k_1} \ge \left( \frac{d^2}{a_0} \right) ^{k_2} \end{aligned}$$

so we have $a_0 (k_2)\le d^2 \Rightarrow a_0 (k_1)\ge a_0 (k_2)$ and $a_0 (k_2)> d^2 \Rightarrow a_0 (k_1)>d^2$. Further,

68 $$\begin{aligned} a_0> d^2 \Rightarrow \left( \frac{d^2}{a_0} \right) ^{k_1} <\left( \frac{d^2}{a_0} \right) ^{k_2} \end{aligned}$$

so $a_0 (k_2)> d^2 \Rightarrow a_0 (k_1) >a_0(k_2)$. Therefore $a_0(k)$ is an increasing function, which means that the bigger the difference between $i_C$ and $i_D$ is, the bigger $a_0$ has to be. This proves that the length of the equilibrium stretch $i_C - i_D$ increases with $a_0$, approaching infinite length as $a_0$ approaches $a^{\prime \prime }_0$. When $a^{\prime \prime }_0<a_0<a^{\prime }_0$ the situation is like the first case, $d^2 \ge bc$ which also has infinite $i_C$, and the interval of equilibria $\llbracket \lceil i_D \rceil , n \rrbracket$ will shrink until it disappears when $a_0 \ge a^{\prime }_0$.

## Appendix 4

Here we prove that $i + \lceil k^{*}(i) \rceil$ is an increasing function of *i*, starting at $\lceil k^{*}(0) \rceil = J_{opt}$ and either ending at $\lceil i_D \rceil$ if $i_D$ exists or continuing to increase for all *i* if $i_D$ does not exist. First, it can be shown that

69 $$\begin{aligned} A(S_{i+k+1},S_i)-A(S_{i+k},S_i) =&~\frac{a_0}{a^2} \left[ A(S_{i+k},S_i)-A(S_{i+k-1},S_i)\right] \nonumber \\&+ \frac{(a_0 -d)(a^2-bc)}{a^2}(bc)^k d^{2i} . \end{aligned}$$

Then, because

70 $$\begin{aligned} \lceil k^{*}(i) \rceil&= \max \left\{ k \ge 1 | A(S_{i+k},S_i) > A(S_{i+k-1},S_i)\right\} \nonumber \\&= \min \left\{ k \ge 1 | A(S_{i+k+1},S_i) < A(S_{i+k},S_i)\right\} , \end{aligned}$$

we have that $\lceil k^{*}(i) \rceil$ increases with *i* if $a_0 >d$, decreases if $a_0 <d$ and is constant if $a_0=d$. As an immediate consequence, we have that $i + \lceil k^{*}(i) \rceil$ increases with *i* for $a_0 \ge d$. We can prove the same is true for $a_0 < d$, in particular for any $i \le \lceil i_D \rceil$ (which we note might be infinite). We fix $i\le \lceil i_D \rceil$ and use $l=i+k$ such that $\lceil i_D \rceil> l >i$. Let $S_j^{(l)}$ be a strategy ending with *j* defections in a subgame of only *l* rounds. We have

71 $$\begin{aligned}&A(S_{l+1},S_{i+1})-A(S_l,S_{i+1})-\left[ A(S_{l+1},S_i)-A(S_l,S_i)\right] \nonumber \\&\qquad = (bc -a^2)a^{2(n-l-1)} \left( A(S^{(l)}_l,S^{(l)}_{i+1})-A(S^{(l)}_l,S^{(l)}_i) \right) \nonumber \\&\qquad = (bc -a^2)a^{2(n-l-1)} \left[ \left( A(S^{(l)}_l,S^{(l)}_{l-1}) - A(S^{(l)}_{l-1},S^{(l)}_{l-1}) \right) \right. \nonumber \\&\qquad \quad + \left. \left( A(S^{(l)}_{l-1},S^{(l)}_{l-1}) - A(S^{(l)}_l,S^{(l)}_{l}) \right) \right] . \end{aligned}$$

The second term in the brackets in (71) is always positive thanks to the diagonal behavior described in Sect. 5.2. The first term in the brackets is also positive, because $l-1 <i_D$, meaning there is a local incentive to defect one more time. Note, we used the fact that $i_D$ is does not depend of the number of rounds in the game (*l* or *n*). To finish the proof, we further note that $\lceil k^{*}(i) \rceil = \max \left\{ k \ge 1 | A(S_{i+k},S_i) < A(S_{i+k-1},S_i)\right\}$.
